# Cx26 drives self-renewal in triple-negative breast cancer via interaction with NANOG and focal adhesion kinase

**DOI:** 10.1038/s41467-018-02938-1

**Published:** 2018-02-08

**Authors:** Praveena S. Thiagarajan, Maksim Sinyuk, Soumya M. Turaga, Erin E. Mulkearns-Hubert, James S. Hale, Vinay Rao, Abeba Demelash, Caner Saygin, Arnab China, Tyler J. Alban, Masahiro Hitomi, Luke A. Torre-Healy, Alvaro G. Alvarado, Awad Jarrar, Andrew Wiechert, Valery Adorno-Cruz, Paul L. Fox, Benjamin C. Calhoun, Jun-Lin Guan, Huiping Liu, Ofer Reizes, Justin D. Lathia

**Affiliations:** 1 Department of Cellular and Molecular Medicine, Lerner Research Institute, Cleveland Clinic, Cleveland, OH 44915 USA; 2 Molecular Medicine, Cleveland Clinic Lerner College of Medicine of Case Western Reserve University, Cleveland, OH 44195 USA; 3 Case Comprehensive Cancer Center, Cleveland, OH 44195 USA; 4 Department of Pathology, School of Medicine, Case Western Reserve University, Cleveland, OH 44106 USA; 5 Departments of Pharmacology and Medicine, Northwestern University School of Medicine, Chicago, IL 60611 USA; 6 Department of Pathology, Cleveland Clinic, Cleveland, OH 44915 USA; 7 Department of Cancer Biology, University of Cincinnati, Cincinnati, OH 45267 USA

## Abstract

Tumors adapt their phenotypes during growth and in response to therapies through dynamic changes in cellular processes. Connexin proteins enable such dynamic changes during development, and their dysregulation leads to disease states. The gap junction communication channels formed by connexins have been reported to exhibit tumor-suppressive functions, including in triple-negative breast cancer (TNBC). However, we find that connexin 26 (Cx26) is elevated in self-renewing cancer stem cells (CSCs) and is necessary and sufficient for their maintenance. Cx26 promotes CSC self-renewal by forming a signaling complex with the pluripotency transcription factor NANOG and focal adhesion kinase (FAK), resulting in NANOG stabilization and FAK activation. This FAK/NANOG-containing complex is not formed in mammary epithelial or luminal breast cancer cells. These findings challenge the paradigm that connexins are tumor suppressors in TNBC and reveal a unique function for Cx26 in regulating the core self-renewal signaling that controls CSC maintenance.

## Abstract (web summary)

Connexin proteins are usually considered as tumor suppressors. Here, the authors show that connexin 26 (Cx26) regulates the self-renewal of breast cancer stem cells via a ternary complex with FAK and NANOG.

## Introduction

Breast cancer remains the leading cause of cancer-related deaths among women worldwide despite advances in screening, diagnosis, and treatment^1,2^. The inter-patient heterogeneity of breast cancer has long been recognized, and molecular genetic approaches have revealed distinct subgroups that are associated with different overall patient outcomes^3,4^. Among these subgroups, triple-negative breast cancer (TNBC), which is defined by the lack of estrogen receptor (ER), progesterone receptor (PR), and Her2/neu receptor expression has the poorest prognosis and accounts for approximately 10% of all breast cancer cases^5,6^. In TNBC, intratumoral cellular heterogeneity has emerged as a hallmark of the malignant state and accounts for persistent tumor growth, therapeutic resistance, and metastasis^3,7,8^. This heterogeneity is considered to be driven at least in part by a self-renewing population of cancer stem cells (CSCs)^9^. The molecular mechanisms of CSC self-renewal are the focus of intensive study as they are likely to yield next-generation therapeutic strategies, as exemplified by the current clinical evaluation of the first generation of anti-CSC therapies^10–12^.

Attempts to elucidate the complexity of CSC maintenance have focused on intrinsic driver mutations and altered developmental signaling pathways^13^. The elevated cellular density within tumors stimulates cellular programs that are activated by cell–cell contact and close proximity. The gap junction (GJ) family of proteins composed of connexin subunits canonically functions in GJ plaques at the interface of adjacent cells to facilitate direct cell–cell communication. Connexins can also function non-canonically as single membrane channels (hemichannels) or as signaling hubs adjacent to any organelle and/or the plasma membrane^14,15^. In the context of cancer, connexins are widely considered to be tumor suppressors in many cancer models^16–19^. However, the prevailing paradigm that connexins have a global tumor-suppressive role has been challenged by emerging evidence showing pro-tumorigenic activities including tumor progression and metastasis^20–23^. In the breast, Cx26 and Cx43 are the predominantly expressed connexin subunits and are implicated in maintaining homeostasis during the development and physiological functioning of the mammary gland^17,24^. In breast cancer, connexins have been described to be both pro- and anti-tumorigenic by regulating transformation, proliferation, cell survival, and metastasis^25,26^. Most studies to date suggest a tumor-suppressive role for Cx26 in early breast cancer progression based on evidence that Cx26 is frequently absent or downregulated in human breast cancer cell lines and human primary tumors^26–28^. However, clinical observations demonstrate a strong correlation between increased Cx26 expression in breast cancer tissue samples harvested following treatment (chemotherapy/surgery) and decreased overall survival^29,30^. Moreover, Cx26 expression has been shown to be associated with increased lymphatic vessel invasion, increased tumor size, and poor prognosis in human breast cancers^29,30^.

While connexin function has been assessed in TNBC, its role in CSCs has yet to be determined. We recently defined a role for Cx46 in CSC maintenance in glioblastoma that opposes the previously described role of Cx43 as a tumor suppressor, suggesting that different connexin family members may play distinct roles^31^. Based on our previous work and observations that Cx26, Cx32, Cx40, and Cx43 do not localize to GJ plaques in breast cancer, we hypothesized that a subset of connexins may regulate CSC maintenance in TNBC independent of their role in cell–cell communication^25,32^. Here, we show that Cx26 forms a complex in TNBC CSCs with the tyrosine kinase focal adhesion kinase (FAK) and the pluripotency transcription factor NANOG to drive CSC maintenance in a GJ-independent manner. Our findings highlight a new function for connexins in the formation of a protein complex that drives CSC self-renewal.

## Results

### Cx26 is expressed in TNBC tissue and CSCs

**Fig. 1:**
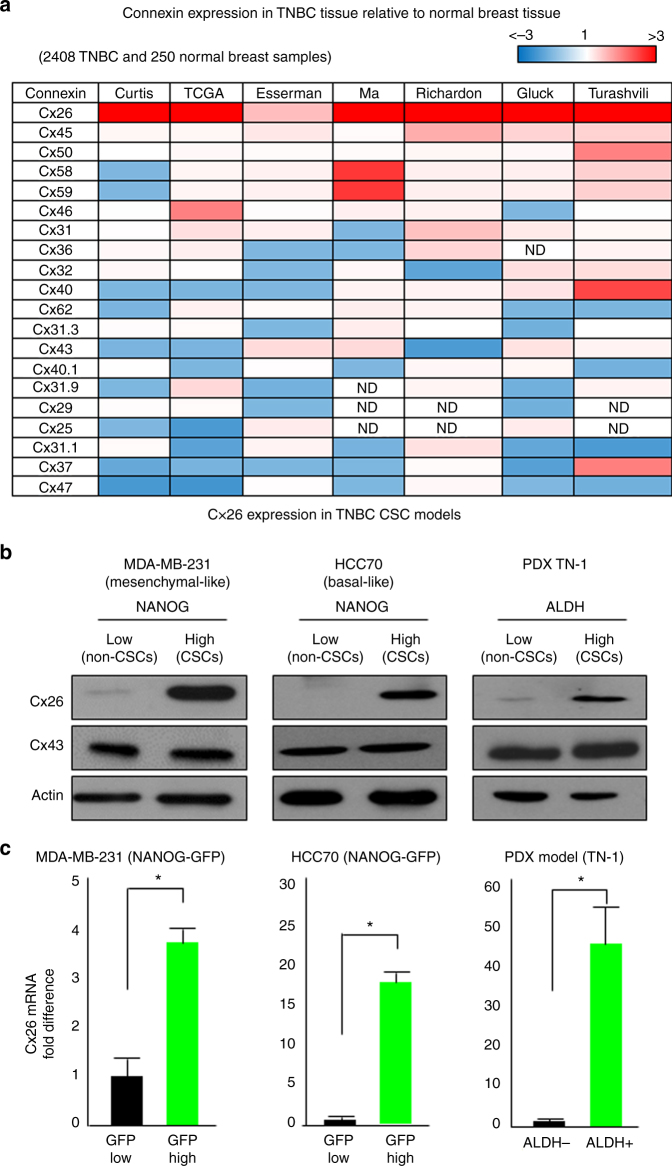
Elevated Cx26 expression in TNBC tissue samples and in TNBC cancer stem cells. **a** Gene expression profiles of 20 different connexins in 2408 TNBC tissue samples were compared with those of 250 normal breast tissue samples across seven different TNBC-normal datasets using the Oncomine^TM^ (Compendia Bioscience, Ann Arbor, MI, USA) database (http://www.oncomine.org/). **b** Cell lysates from CSCs enriched from two TNBC cell lines of different subtypes and from a TNBC PDX were probed with anti-Cx26 and anti-Cx43 antibodies. The NANOG-GFP reporter was used to enrich CSCs from MDA-MB-231 (mesenchymal-like) and HCC70 (basal-like) cells, and PDX TN-1 CSCs were enriched according to ALDH activity. Actin was used as a loading control. **c** mRNA expression was determined by qPCR and compared between cancer stem cells (CSCs) and non-CSCs enriched from MDA-MB-231 and HCC70 cells using the NANOG-GFP reporter system and between those enriched from PDX TN-1 cells using ALDH activity sorting (ALDEFLUOR assay). Actin was used as a normalization control (*p < 0.05). All the error bars indicate standard deviation. ND, not determined

To investigate the role of connexins in the context of their previously reported role as tumor suppressors, we interrogated 7 different datasets containing a total of 250 normal breast samples and over 2,400 TNBC samples and found that *Cx26* was the most highly expressed connexin in TNBC versus non-neoplastic mammary gland tissue (Fig. 1a). This is distinct from the low levels of *Cx26* seen in cultured triple-negative MDA-MB-231 breast cancer cells^24,33^ and previous observations that some connexins displayed lower expression in breast cancer tissue^28^. Given the elevated expression of *Cx26* in TNBC tissue and the cellular heterogeneity present within these tumors, we assessed whether Cx26 expression was elevated in a specific tumor cell population, including CSCs. To address this possibility, we enriched CSC populations using a CSC reporter system (NANOG promoter-driven green fluorescent protein (GFP)) in two established TNBC cell line models we previously developed^34^ and using the ALDEFLUOR assay based on aldehyde dehydrogenase (ALDH) activity^35^ in a TNBC patient-derived xenograft (PDX) model^36,37^. In all three models, the CSC-enriched population expressed significantly higher levels of Cx26 compared to non-CSCs at the protein level using immunoblot analysis (Fig. 1b). Likewise, the CSC-enriched population expressed significantly higher levels of *Cx26* mRNA compared with non-CSCs (Fig. 1c). To control for connexin specificity, Cx43 expression in CSCs and non-CSCs was immunoblotted, and we observed no difference in expression between CSCs and non-CSCs (Fig. 1b). These results demonstrate that Cx26 is elevated in TNBC CSCs.

### Cx26 is important for CSC maintenance

**Fig. 2:**
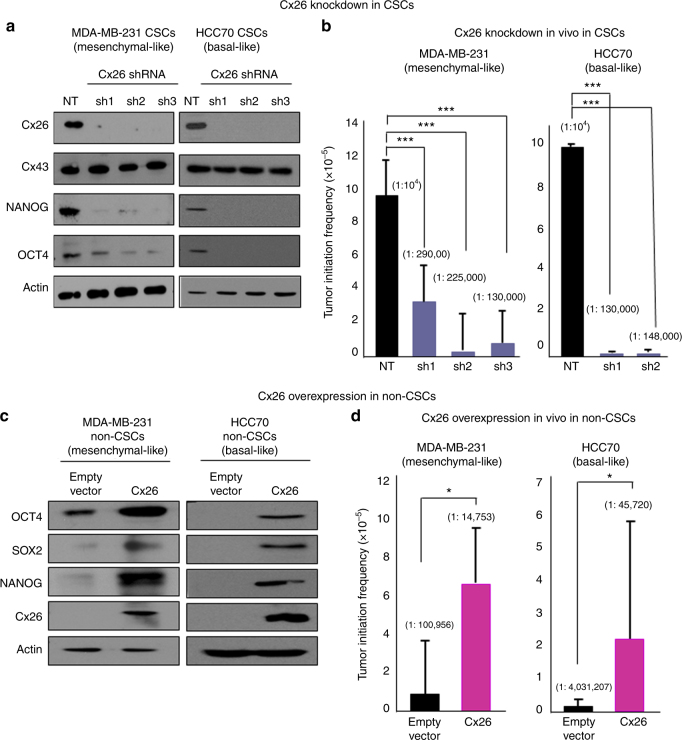
Cx26 is necessary and sufficient for the maintenance of self-renewal, in vivo tumor initiation, and NANOG expression. **a** Cell lysates from MDA-MB-231 and HCC70 CSCs silenced using three Cx26 shRNA constructs (sh1, sh2, and sh3) and a non-targeting shRNA (NT) control were probed with Cx26, Cx43, NANOG, and OCT4 antibodies. Actin was used as a loading control. **b** In vivo tumor initiation studies were performed in *Cx26*-silenced MDA-MB-231 and HCC70 CSCs with at least four mice per group, and the *p* value was calculated using a log-rank analysis. The graphs show the estimates of stem cell frequencies of NT control compared with the *Cx26* shRNA silencing constructs and their corresponding *p* values. **c** MDA-MB-231 and HCC70 non-CSCs containing Cx26 overexpression vector or empty vector were probed with anti-Cx26, NANOG, OCT4, and SOX2 antibodies. Actin was used as a loading control. **d** In vivo tumor initiation studies were performed comparing the empty vector group with the Cx26 overexpression group, and the *p* value was calculated using a log-rank analysis. The graphs show the estimates of stem cell frequencies with the corresponding *p* values for the empty vector compared with Cx26 overexpression in MDA-MB-231 and HCC70 non-CSCs. (*p < 0.05, ***p < 0.001). All the error bars indicate the range between the upper and median levels

To determine the functional significance of Cx26 in CSCs, we employed a genetic approach to attenuate *Cx26* expression. Using three separate non-overlapping Cx26 short hairpin RNA (shRNA) constructs, we inhibited Cx26 protein expression in MDA-MB-231 and HCC70 CSCs without altering the levels of Cx43, which in other systems has been shown to be induced to compensate for the loss of Cx26 (Fig. 2a)^38^. We found that silencing of *Cx26* decreased the expression of NANOG, a key transcription factor important for CSC phenotypes^39^ (Fig. 2a). To assess whether *Cx26* silencing altered the self-renewal capacity of MDA-MB-231 CSCs, we performed limiting dilution tumorsphere-formation analysis. *Cx26*-silenced CSCs exhibited significantly lower self-renewal capacity and hence were estimated to have lower stem cell frequencies compared to control CSCs transduced with a non-targeting (NT) shRNA (reduced from 1 in 16 in NT conditions to 1 in 31.5, 1 in 33.1, and 1 in 38 in the three *Cx26* shRNA conditions; Supplementary Fig. 1a). To validate the effect of *Cx26* suppression on tumor initiation capacity, we performed an in vivo limiting dilution analysis. *Cx26*-silenced MDA-MB-231 and HCC70 CSCs exhibited significantly lower tumor initiation frequency compared to control NT CSCs (in MDA-MB-231 CSCs, frequency was reduced from 1 in 10,000 in non-target conditions to 1 in 290,000, 1 in 225,000, and 1 in 130,000 in the three *Cx26* shRNA conditions; in HCC70 CSCs, frequency was reduced from 1 in 10,000 in non-target conditions to 1 in 130,000 and 1 in 148,000 in the two *Cx26* shRNA conditions; Fig. 2b and Supplementary Fig. 1e). These data demonstrate that Cx26 is important for CSC maintenance and tumor initiation.

### Cx26 is sufficient to drive CSC phenotypes in non-CSCs

Based on the elevation of Cx26 in CSCs and its essential role in CSC maintenance, we assessed whether Cx26 elevation was sufficient to induce expression of pluripotency genes in non-CSCs, which express low levels of Cx26. We took advantage of our NANOG promoter-driven GFP reporter system that allows for the direct visualization of the stem cell phenotype using a readout of GFP signal^34^. We introduced Cx26 into MDA-MB-231 GFP-negative and HCC70 GFP-negative non-CSCs and found that these cells exhibited an increase in OCT4, SOX2, and NANOG protein expression (Fig. 2c). The overexpression of Cx26 in MDA-MB-231 non-CSCs induced a significant increase in self-renewal and stem cell frequency (from 1 in 58.8 with the empty vector to 1 in 27 with Cx26 overexpression) as assessed by limiting dilution sphere-formation analysis (Supplementary Fig. 1b). We observed an increase in GFP signal in MDA-MB-231 and HCC70 non-CSCs transduced with Cx26 compared with the empty vector as an independent reporter of NANOG induction (Supplementary Fig. 1c). In contrast, Cx26 overexpression in luminal breast cancer (MCF7) cells showed no significant increase in sphere-initiating frequency compared with the empty vector (Supplementary Fig. 1d). Moreover, Cx26 overexpression in MDA-MB-231 and HCC70 non-CSCs resulted in significantly elevated tumor initiation frequency compared with empty vector (in MDA-MB-231 non-CSCs, frequency increased from 1 in 100,956 for cells transduced with empty vector compared to 1 in 14,753 for the cells overexpressing Cx26; in HCC70 non-CSCs, frequency increased from 1 in 4,031,207 for cells transduced with empty vector compared to 1 in 45,720 for the cells overexpressing Cx26; Fig. 2d and Supplementary Fig. 1e). These data demonstrate that driving Cx26 expression in non-CSCs is sufficient to induce CSC marker expression and increase self-renewal and tumor initiation.

### Cytoplasmic and nuclear localization of Cx26 in TNBC

Based on previous reports showing limited GJ-dependent coupling in MDA-MB-231 TNBC cells^33^, we assessed whether dye transfer could be detected in our TNBC CSC system. Using a single-cell microinjection approach^31^, we observed limited biocytin-rhodamine dye transfer between CSCs (Supplementary Movie 1), confirming previous results and suggesting that TNBC cells may not utilize connexins for GJ-mediated cell–cell communication^33^. Connexins have been shown to possess channel-independent functions, although these have not been extensively explored for Cx26^40,41^.

**Fig. 3:**
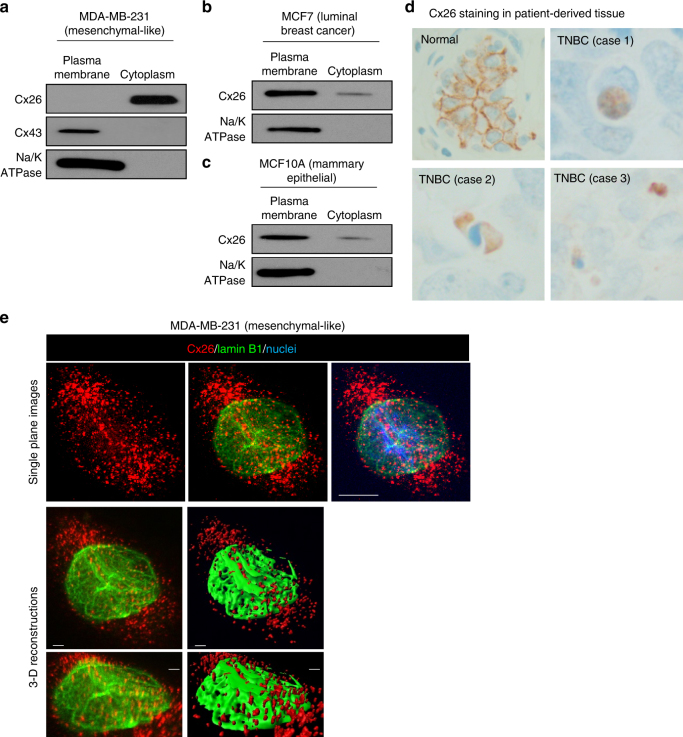
Localization of Cx26 in MDA-MB-231, MCF7, and MCF10A cells. **a** The cytosol and organelle fraction and the plasma membrane fraction of MDA-MB-231 parental cells were probed for a plasma membrane marker (sodium potassium ATPase), Cx26, and Cx43 by immunoblotting. **b**, **c** Cx26 localization in MCF7 and MCF10A cells was determined by immunoblotting of the plasma membrane and the cytosol and organelle fractions with Na/K ATPase as an indicator of plasma membrane fraction quality. **d** Immunohistochemistry micrographs demonstrate the staining pattern of Cx26 (brown) in normal and adjacent TNBC tissue from three patients. Nuclei were counterstained in blue. Images provided at 400×. **e** Confocal micrographs of MDA-MB-231 TNBC cells stained with antibodies against Cx26 (red) and lamin B1 (green). Single plane images and 3-D reconstructions of areas were generated in Imaris. Corresponding fluorescent images are provided above 3-D reconstructions. Nuclei were counterstained with Hoechst 33342, and scale bars are provided on each micrograph and reconstruction

As previous work demonstrated that Cx26 is not localized to the plasma membrane in TNBC cells and we found limited GJ function in Cx26-expressing TNBC cells, we assessed the localization of Cx26 in our cells^33,42^. We performed subcellular fractionation analyses of MDA-MB-231 cells and observed that Cx26 was enriched in the cytoplasmic fraction (Fig. 3a). While Cx26 was not expressed at a detectable level in the plasma membrane fraction, Cx43 was present at the plasma membrane as expected (Fig. 3a). We further investigated the localization of Cx26 in MCF7 and MCF10A luminal breast cancer and mammary epithelial cells and determined that Cx26 was predominantly localized in the isolated plasma membranes of both lines compared with the cytoplasmic fraction (Fig. 3b, c). To confirm these findings, we performed immunohistological staining for Cx26 in TNBC patient-derived pathological specimens compared to adjacent normal mammary gland tissue. While Cx26 was localized to the plasma membrane in normal mammary epithelium, Cx26 displayed an intracellular localization in three distinct TNBC patient specimens (Fig. 3d). We next performed immunofluorescence analysis followed by confocal microscopy to visualize the subcellular localization of Cx26. In MDA-MB-231 and HCC70 cells, Cx26 was found in the cytoplasm and associated with the nuclear envelope based on co-staining with lamin B1, a nuclear envelope protein (Fig. 3e and Supplementary Fig. 2b, c). Nuclear localization of connexins has been previously described^15,43^. In mammary epithelial cells, Cx26 was localized at the plasma membrane (Supplementary Fig. 2a), consistent with the cellular fractionation analysis (Fig. 3c). Collectively, these data indicate that in TNBC, Cx26 is not expressed in the plasma membrane where connexins form GJs or connexons. These data support a GJ-independent role of Cx26 that is critical to maintain self-renewal and tumor initiation capacity in TNBC CSCs.

### Cx26 is present in a complex containing NANOG and FAK

Our observations indicated that Cx26 was localized adjacent to the nucleus and that *Cx26* silencing via shRNA attenuated NANOG expression in CSCs, while Cx26 overexpression increased NANOG expression in non-CSCs. We next assessed whether Cx26 could interact either directly or indirectly with NANOG.

As we were not able to find any previously reported direct interaction between Cx26 and NANOG, we focused on known connexin-interacting partners that also interact with NANOG, and FAK emerged as a candidate^44^. FAK is known to interact with Cx26 and, interestingly, also interacts with NANOG outside the nucleus to phosphorylate NANOG^45,46^. In addition, FAK has been demonstrated to be essential for TNBC CSC maintenance^47^, and FAK autophosphorylation at Y397 is an essential event in stem cell self-renewal^48^. We investigated the role of FAK in TNBC cells by silencing *FAK* expression in CSCs or by overexpressing FAK in non-CSCs. *FAK* silencing attenuated the self-renewal of CSCs (Supplementary Fig. 3a), confirming previous reports^47^, while FAK overexpression induced self-renewal in non-CSCs (Supplementary Fig. 3b) as measured by sphere-formation assays. FAK function is regulated by a series of autophosphorylation events, some of which have been linked to self-renewal^49^. Therefore, we assessed FAK phosphorylation status in CSCs and non-CSCs enriched using our NANOG promoter-driven GFP reporter. Compared with non-CSCs, CSCs contained higher levels of phosphorylation at FAK residue Y397, the autophosphorylation site important for self-renewal (Supplementary Fig. 3c), while other sites of FAK were phosphorylated to similar levels in the two populations^47,49^.

**Fig. 4:**
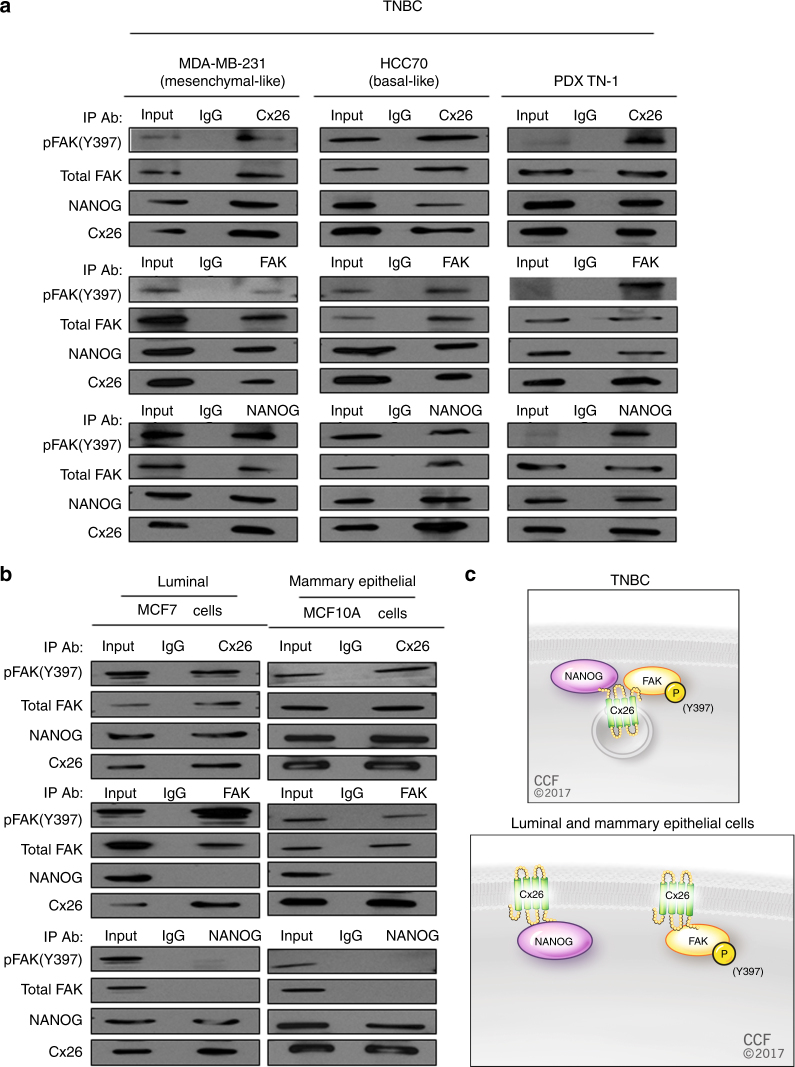
Cx26 forms a TNBC-specific complex with focal adhesion kinase (FAK) and NANOG. Cell lysates from bulk cell cultures of **a** mesenchymal-like TNBC (MDA-MB-231), basal-like TNBC (HCC70), TNBC (PDX TN-1), and **b** non-TNBC (MCF7 and MCF10A) were subjected to immunoprecipitation with anti-Cx26, anti-FAK, and anti-NANOG antibodies. pFAK (Y397), FAK, Cx26, and NANOG proteins in the precipitated complex were detected by western blotting using specific antibodies. Fifteen percent of the lysate used for immunoprecipitation was loaded as the input control. As a negative control, immunoprecipitation with the corresponding non-immune IgG was performed. **c** Schematic summarizing the interactions detected in TNBC versus mammary epithelial and luminal breast cancer cells

To determine whether Cx26, NANOG, and FAK physically interact, we performed immunoprecipitation studies in a diverse set of TNBC cells (Fig. 4a and Supplementary Fig. 4). We analyzed two mesenchymal-like lines (MDA-MB-231 and MDA-MB-157), three basal-like lines (HCC38, HCC70, and MDA-MB-468), and cells from a PDX (TN-1). We also analyzed a luminal breast cancer line (MCF7) and mammary epithelial cells (MCF10A) (Fig. 4b). In all the TNBC lines we analyzed, both FAK and NANOG were co-immunoprecipitated with Cx26 (Fig. 4a and Supplementary Fig. 4). To confirm whether these three proteins exist in a complex, we performed immunoprecipitation using a specific antibody against NANOG or FAK and examined which partners were co-precipitated. In TNBC cells, a FAK antibody co-precipitated both Cx26 and NANOG, while a NANOG antibody likewise co-precipitated Cx26 and FAK (Fig. 4a and Supplementary Fig. 4). However, in MCF7 and MCF10A cells, when FAK was immunoprecipitated, NANOG was barely detected, and when NANOG was immunoprecipitated, FAK and pFAK (Y397) were not detected in the immunoprecipitate (Fig. 4b). We confirmed that Cx26, NANOG, and FAK proteins were expressed at similar levels in all analyzed cells, precluding the possibility that the differences in complex formation were due to differences in protein expression between TNBC, luminal breast cancer, and mammary epithelial cells (Supplementary Fig. 5). These data indicate that both NANOG and FAK interact with Cx26 in TNBC cells but that the NANOG/FAK interaction is not robust in MCF7 or MCF10A cells (Fig. 4c).

Based on our observation that Cx26 is located in the cytoplasmic and nuclear fractions of MDA-MB-231 cells, we next assessed whether the ternary protein complex was present in both cellular fractions. Immunoprecipitation experiments of the fractions using anti-FAK antibody detected the presence of both Cx26 and NANOG in both the cytoplasmic and nuclear fraction immunoprecipitates (Supplementary Fig. 6a, b), indicating that the FAK ternary complex is present in both the nuclear and cytoplasmic compartments.

### *Cx26* expression regulates NANOG stability

**Fig. 5:**
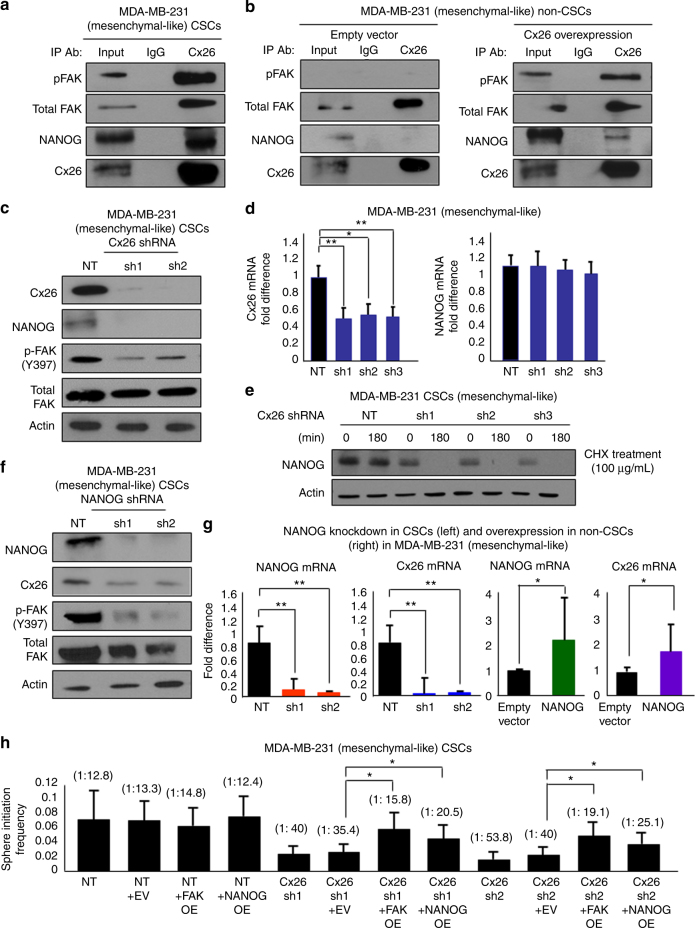
The Cx26/NANOG/FAK interaction is enriched in MDA-MB-231 CSCs, and Cx26 regulates NANOG protein stability in MDA-MB-231 CSCs. **a** Immunoprecipitation with anti-Cx26 antibody was performed in MDA-MB-231 CSCs and **b** non-CSCs transduced with either empty vector or Cx26 overexpression vector. The precipitates were probed for pFAK (Y397), total FAK, NANOG, and Cx26 by immunoblotting. **c** Immunoblots of the cell lysates of MDA-MB-231 NANOG-GFP CSCs silenced for Cx26 using two shRNA constructs each (sh1 and sh2) and a non-targeting (NT) control were probed with Cx26, NANOG, pFAK (Y397), and total FAK antibodies. Actin was used as a loading control. **d** Fold difference in mRNA expression of *Cx26* and *NANOG* in *Cx26*-silenced MDA-MB-231 CSCs compared with NT control was determined by qPCR. Actin was used as a normalization control (*p < 0.05, **p < 0.01). Error bars indicate standard deviation. **e** *Cx26*-silenced and non-targeting control (NT) MDA-MB-231 CSCs were treated with cycloheximide (CHX) to block de novo protein synthesis. Cells were harvested at 0 and 180 min following cycloheximide treatment and probed for NANOG expression by immunoblotting. Actin was used as a loading control. **f** Immunoblots of MDA-MB-231 NANOG-GFP CSCs silenced for *NANOG* using two shRNA constructs each and a non-targeting control were probed with Cx26, NANOG, pFAK (Y397), and total FAK antibodies. Actin was used as a loading control. **g** Fold difference in mRNA expression of *Cx26* and *NANOG* in *NANOG*-silenced MDA-MB-231 CSCs and NANOG-overexpressed MDA-MB-231 non-CSCs compared with their corresponding controls was determined by qPCR. Actin was used as a normalization control (*p < 0.05, **p < 0.01). Error bars indicate standard deviation. **h** Stem cell frequencies of *Cx26*-silenced MDA-MB-231 CSCs overexpressing FAK or NANOG compared with NT and/or empty vector controls were determined by limiting dilution sphere-forming assays. (*p < 0.05). The error bars indicate the range between the upper and median levels

Based on our observation that Cx26, NANOG, and FAK interact with each other in TNBC cells, we confirmed that the Cx26/NANOG/FAK complex was present along with an enrichment of autophosphorylated pFAK (Y397) in MDA-MB-231 CSCs (Fig. 5a) and HCC70 CSCs (Supplementary Fig. 7a). In contrast, the ternary complex could not be detected in MDA-MB-231 non-CSCs (Fig. 5b) and HCC70 non-CSCs (Supplementary Fig. 7b). However, overexpression of Cx26 in MDA-MB-231 non-CSCs was sufficient to drive formation of the complex (Fig. 5b; and in HCC70 non-CSCs, Supplementary Fig. 7b). Moreover, Cx26 overexpression in MDA-MB-231 non-CSCs resulted in a parallel activation of FAK based on autophosphorylation at Y397 (pFAK, Fig. 5b; and in HCC70 non-CSCs, Supplementary Fig. 7b).

To further assess the interaction between Cx26, NANOG, and FAK, we silenced the individual components in MDA-MB-231 CSCs and assessed overall expression levels of the other members. Silencing *Cx26* in MDA-MB-231 CSCs inhibited NANOG protein expression and FAK autophosphorylation (Y397), but did not affect total FAK levels (Fig. 5c; and in HCC70, Supplementary Fig. 7c) or the ability of FAK to be immunoprecipitated by FAK antibodies (Supplementary Fig. 8). As Cx26 decreased the protein levels of NANOG, we examined the mechanism of NANOG suppression. *NANOG* mRNA expression was not inhibited in *Cx26*-silenced MDA-MB-231 CSCs compared to NT controls (Fig. 5d; and in HCC70 CSCs, Supplementary Fig. 7d), indicating that post-transcriptional regulation is predominantly responsible for the decrease in NANOG protein levels. As a control, we assessed *Cx26* expression and found that *Cx26* mRNA was decreased in *Cx26*-silenced MDA-MB-231 CSCs compared to NT controls (Fig. 5d; and in HCC70 CSCs, Supplementary Fig. 7d). As altering Cx26 did not greatly impact the *NANOG* transcript level, we next assessed whether NANOG protein stability was decreased by blocking protein synthesis with 100 μg/mL cycloheximide (CHX). *Cx26*-silenced MDA-MB-231 CSCs exhibited a faster decline in NANOG protein levels compared to NT controls (Fig. 5e; and in HCC70 CSCs, Supplementary Fig. 7e). These results demonstrate that *Cx26* expression is important for maintaining NANOG protein stability.

*NANOG* silencing decreased the levels of both Cx26 and total FAK in MDA-MB-231 CSCs (Fig. 5f) and HCC70 CSCs (Supplementary Fig. 9a). NANOG regulated *Cx26* at the transcriptional level, as shRNA silencing of *NANOG* led to decreased *Cx26* mRNA in MDA-MB-231 CSCs (Fig. 5g) and HCC70 CSCs (Supplementary Fig. 9b). Likewise, NANOG overexpression led to increased *Cx26* mRNA in MDA-MB-231 non-CSCs (Fig. 5g) and HCC70 non-CSCs (Supplementary Fig. 9c). In contrast, silencing *FAK* inhibited expression of Cx26 protein but did not attenuate NANOG protein expression (Supplementary Fig. 10a). As autophosphorylation of FAK was decreased by silencing *Cx26* and enhanced by Cx26 overexpression, we tested whether FAK autophosphorylation was required for complex formation. We performed lentiviral transduction of hemagglutinin (HA)-tagged wild-type (WT) FAK or a FAK mutant (Y397F) into MDA-MB-231 cells followed by immunoprecipitation with HA antibody. We determined that both WT and mutant FAK (Y397F) were co-immunoprecipitated in complex with NANOG and Cx26 (Supplementary Fig. 10b). These data support the hypothesis that the members of the Cx26/FAK/NANOG complex can regulate each other, with Cx26 regulating NANOG protein stability and FAK activation.

We next tested whether NANOG or FAK could functionally complement the inhibition of sphere-initiating frequency in *Cx26*-silenced cells. In *Cx26*-silenced MDA-MB-231 CSCs, both NANOG and FAK were able to individually increase sphere-initiating frequency, while empty vector did not (Fig. 5h). Likewise, Cx26-induced sphere initiation frequency in MDA-MB-231 non-CSCs could be attenuated by shRNA silencing of either *FAK* or *NANOG* (Supplementary Fig. 11). These rescue studies suggest that NANOG and FAK are able to functionally complement the changes in self-renewal induced by Cx26.

### Cx26/NANOG/FAK complex is necessary for self-renewal

Our data indicate that Cx26 interacts with NANOG and FAK in a ternary complex that is localized adjacent to the nucleus and that introduction of Cx26 is sufficient to drive complex formation. To further assess the dynamics of ternary complex formation, we assessed the effect of altering Cx26 localization using two loss-of-function point mutants, D66H-Cx26 and G59A-Cx26. These mutations have been characterized in the context of sensorineural deafness and hyper-proliferative skin disorders^50,51^ and fail to traffic out of the Golgi apparatus^52^, allowing us to test whether mislocalization of Cx26 affects its interaction with NANOG and FAK. When we introduced a red fluorescent protein (RFP)-tagged version of D66H-Cx26 into CSCs, the majority of the RFP signal was co-localized with the Golgi apparatus marker GM-130, confirming its mislocalization (Supplementary Fig. 12). RFP-tagged WT Cx26, in contrast, was found in both the Golgi apparatus and the nucleus, an expression pattern similar to that of the endogenous Cx26 protein (Supplementary Fig. 12).

**Fig. 6:**
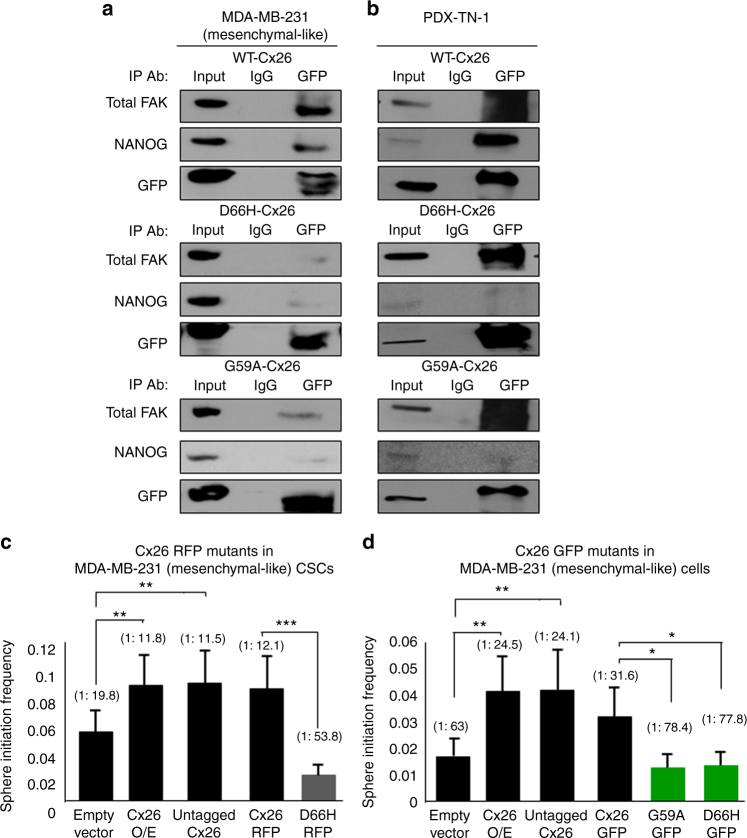
Cx26 mutants failing to complex with NANOG or FAK/NANOG disrupt the self-renewal capacity of TNBC CSCs. **a**, **b** After transfection of plasmids expressing GFP-fused Cx26 wild-type and mutant proteins, immunoprecipitation was performed using a GFP antibody in MDA-MB-231 and PDX TN-1 cells, and the resulting immunoprecipitates were probed with FAK, NANOG, and GFP antibodies. **c** Stem cell frequencies determined by limiting dilution sphere-forming assays indicate that the expression of the D66H-RFP mutant Cx26 in MDA-MB-231 CSCs significantly reduced the stem cell frequency compared with the expression of the wild-type Cx26-RFP and empty vector control. **d** D66H-GFP or G59A-GFP mutant Cx26 expression in MDA-MB-231 parental cells failed to increase stem cell frequency compared with the expression of the wild-type Cx26-GFP (*p < 0.05, **p < 0.01, ***p < 0.001). All the error bars indicate the range between the upper and median levels

**Fig. 7:**
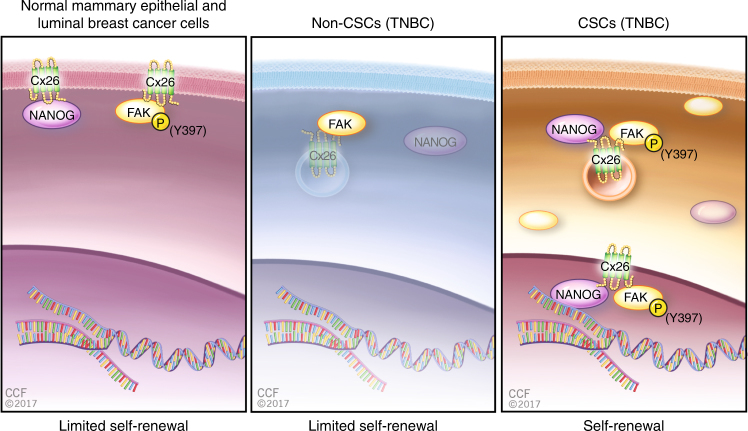
Model of Cx26, NANOG, and FAK interaction in luminal breast cancer and TNBC cells. Schematic depicting the interaction between Cx26, NANOG, and FAK. In luminal breast cancer cells, Cx26 interacts with FAK and NANOG individually, but the Cx26/NANOG/FAK complex does not form. In TNBC non-CSCs, FAK interacts with Cx26 but is not phosphorylated and NANOG is not present in a complex with Cx26 and FAK. In TNBC CSCs, all three proteins are expressed and form a complex, driving self-renewal

To analyze the ability of each Cx26 mutant to complex with FAK and NANOG, we introduced GFP-tagged versions of WT, G59A, or D66H mutant Cx26 into parental MDA-MB-231 cells expressing no GFP. Immunoprecipitation using a GFP antibody co-precipitated NANOG and FAK from MDA-MB-231 cells expressing WT Cx26-GFP, as expected (Fig. 6a), and this observation was reproduced in PDX cells (Fig. 6b). However, when D66H or G59A mutant Cx26-GFP was introduced into MDA-MB-231 or PDX cells, immunoprecipitation using a GFP antibody co-precipitated FAK but not NANOG, indicating that these mutants fail to complex with NANOG (Fig. 6a, b) and demonstrating that mislocalized Cx26 has a limited ability to form the ternary complex. We next assessed the biological consequences of the mislocalized Cx26 mutants on sphere initiation frequency. When the Cx26 D66H mutant was introduced into MDA-MB-231 CSCs, we observed reduced sphere initiation frequency (1 in 19.8 for empty vector, 1 in 11.8 for Cx26 overexpression, 1 in 11.5 for untagged Cx26, 1 in 12.1 for Cx26-RFP, and 1 in 53.8 for D66H-RFP, Fig. 6c), indicating that self-renewal was disrupted. Further, when Cx26 D66H and G59A mutants were introduced into MDA-MB-231 parental cells, sphere initiation frequency was not enhanced compared to introduction of WT Cx26 (1 in 63 for empty vector, 1 in 24.5 for Cx26 overexpression, 1 in 24.1 for untagged Cx26, 1 in 31.6 for Cx26-GFP, 1 in 78.4 for G59A-GFP, and 1 in 77.8 for D66H-GFP; Fig. 6d). Collectively, these data indicate that Cx26 mutations suppress NANOG-binding activity and also abrogate the ability of Cx26 to promote sphere formation, highlighting the critical role of Cx26 in the formation of a ternary complex that promotes CSC maintenance (Fig. 7).

### A *Cx26/NANOG/FAK* signature predicts poor prognosis in TNBC

Our observations demonstrate that a ternary complex containing Cx26, NANOG, and FAK plays an essential role in TNBC CSC self-renewal and tumor initiation capacities. Based on this finding, we hypothesized that a gene expression signature of all three of these genes would predict TNBC patient prognosis. To test this hypothesis, we assessed *Cx26* mRNA expression across multiple breast cancer subtypes using the breast cancer gene expression miner (version 4.0; http://bcgenex.centregauducheau.fr/). We did not observe a significant difference in *Cx26* expression across datasets, with the exception of progesterone receptor (PR)-positive tumors having significantly more *Cx26* as compared to PR-negative tumors (Supplementary Fig. 13a). As such, we expanded our survival analysis using the Kaplan–Meier (KM) plotter (http://kmplot.com/) and found that *Cx26* did not predict significant differences in relapse-free survival in any breast cancer subtype (Supplementary Fig. 13b). However, we did observe significant differences in relapse-free survival when we combined *Cx26* with *FAK* and *NANOG* in TNBC, the specific subtype where the interaction of all three proteins was detected (Supplementary Fig. 13c and Supplementary Table 1). Collectively, these analyses indicate that *Cx26* expression may be uniform across breast cancer subtypes, excluding the PR-positive subtype, but that when *FAK* and *NANOG* are expressed together with *Cx26*, the permissive cellular environment of TNBC cells allows the formation of a Cx26/FAK/NANOG complex that may drive CSC maintenance by activating self-renewal.

## Discussion

Our studies reveal a new GJ-independent role for Cx26 in the maintenance of CSC self-renewal in TNBC. Connexins are predominantly considered tumor suppressors in many solid cancer models^53,54^; however, recent studies challenge this paradigm, as connexins appear to modulate invasion and metastasis, indicating that they are only conditional tumor suppressors. We demonstrate that Cx26 is present in a ternary complex with FAK and NANOG in the cytoplasm and adjacent to the nucleus. Moreover, we find that FAK activation and NANOG stability are dependent on Cx26 in TNBC. This complex is specific to TNBC, as NANOG and FAK do not co-immunoprecipitate in mammary epithelial and luminal breast cancer cells. Complex formation with Cx26 may provide a scaffold for the NANOG/FAK interaction that is critical for self-renewal specifically in TNBC, as depicted in our working model (Fig. 7).

Self-renewal programs ensure the maintenance of a reservoir of stem cells that can give rise to differentiated progeny. Intrinsic programs and extrinsic factors (such as mitogens and cell–cell interactions) play pivotal roles in determining the fate of CSCs (self-renewal versus differentiation). We observed that NANOG and FAK, two intrinsic factors that are critical for breast CSCs, physically interact with Cx26 and that the expression of Cx26 was essential for Y397 phosphorylation of FAK and the stability of NANOG. Additional functional studies demonstrated that *Cx26* expression did not control *NANOG* transcription; however, *Cx26* mRNA levels appear to be linked to NANOG levels. This may explain the elevated levels of *Cx26* mRNA and protein in CSCs; however, there is likely to be additional levels of regulation. NANOG also appears to control both Cx26 and FAK protein levels, while FAK likely controls Cx26 but not NANOG protein levels. Taken together, these data suggest that Cx26 serves as a critical signaling hub via its interaction with NANOG and FAK^44–46^ and reveal a complex regulatory network among Cx26, NANOG, and FAK.

There is building evidence that the NANOG signaling network is expansive and interacts with proteins that have functions beyond stem cell maintenance. NANOG downstream regulation is relatively well characterized, and emerging evidence suggests that NANOG can interact with other proteins such as Bmi1 and Numb via aurora A kinase and atypical protein kinase C zeta to promote self-renewal and tumorigenesis^55–57^. The regulation of NANOG expression itself in cancer requires a more in-depth understanding. In embryonic stem cells, NANOG degradation is regulated by a motif rich in proline, glutamine, serine, and threonine, and NANOG protein stability has been described to require interaction with developmental pluripotency-associated 5 or Pin1 via NANOG phosphorylation^58–60^. Our findings suggest that the NANOG signaling network can be influenced by connexins in a GJ-independent manner that results in stabilization of NANOG. Likewise, the role of FAK has expanded beyond its well-established function in cell adhesion. For example, FAK directly phosphorylates NANOG to promote cell survival and to induce self-renewal^45,46^.

Breast cancer has served as an experimental paradigm for molecular analysis and characterization of CSCs, serving as a precursor to The Cancer Genome Atlas efforts^61^ and representing the first tumor to be subclassified and the first solid tumor found to contain CSCs^9^. CSCs represent a barrier to the development of more effective TNBC therapies. Recent single-cell data from primary breast tumors indicate that cell lines can be used as relevant model systems to interrogate the functions of CSCs, as high similarities between cell lines and primary breast cancer samples have been observed^61^. This further strengthens our findings using our TNBC CSC reporter system and the relevance of this system for revealing a novel interaction among Cx26, NANOG, and FAK that may be amenable for clinical targeting. Despite its importance in self-renewal, targeting NANOG for therapeutic purposes remains difficult as it is a transcription factor. FAK, on the other hand, is a candidate for targeting, as FAK inhibitors have been developed and are currently being evaluated in the context of tumor initiation, growth, and metastasis^49^. However, FAK is essential in numerous normal cellular processes, and while FAK inhibition may alter NANOG activity, it is likely to be associated with adverse clinical complications^62–64^. Our current study suggests that targeting Cx26 may be an effective alternative to targeting both FAK and NANOG, as modulating Cx26 expression and function disrupt the critical Cx26/NANOG/FAK ternary complex that is vital for non-TNBC tumorigenicity. It remains unclear whether the ternary complex is formed in TNBC CSCs due to higher Cx26 levels, as we observed that overexpression was sufficient to form the Cx26/NANOG/FAK complex, or if the CSC phenotype was responsible for complex formation. Interrogating these potential mechanisms via methods to specifically disrupt complex initiation and stability represents the focus of future studies, as this may provide insight that could be leveraged for therapeutic development. It also remains unclear as to why the Cx26/NANOG/FAK complex may be a unique feature of TNBC, given that all three proteins are expressed in other breast cancer cells and mammary epithelial cells. One hypothesis for further testing is that the mislocalization of Cx26 away from the plasma membrane can be leveraged for scaffolding of proteins, including NANOG and FAK, and this serves as a mechanism to ensure function and decrease protein degradation. Further, NANOG function may be enhanced due to the interaction with other complex members or that its target genes may be different between complex-bound NANOG and NANOG located outside this complex represents an unexplored mechanism unmasked by our findings. Finally, a therapeutic angle for consideration may be inhibiting Cx26 in the context of metastatic disease, as Cx26 has been shown to enhance metastasis, likely by promoting lymphatic vessel invasion^26,29^. Targeting Cx26 may have less adverse complications than targeting FAK, as Cx26 is essential for the mammary epithelium during early pregnancy but is not essential for mammary cell function^37,65^. However, adverse effects on cochlear hair cells must be carefully examined. A *Cx26/NANOG/FAK* signature might also be clinically useful as a new prognostic factor informative of TNBC patient outcome. Taken together, our studies reveal a unique signaling complex containing Cx26, NANOG, and FAK that may be amenable for targeting and compromising TNBC CSC maintenance.

## Methods

### Cell culture

MDA-MB-231, HCC38, MDA-MB-157, MDA-MB-468, MCF10A, MCF7 and HCC70 breast cancer cells and HEK293T cells (American Type Culture Collection, Manassas, VA, USA) were cultured in log-growth phase in modified Eagle’s medium (MEM)/Dulbecco’s modified Eagle’s medium (DMEM/mammary epithelial cell growth medium; Lonza, Basel, Switzerland) for MCF10A, supplemented with 1 mM sodium pyruvate (Cellgro, Kansas City, MO, USA) and 10% heat-inactivated fetal calf serum (FCS) at 37 °C in a humidified atmosphere (5% CO_2_). Triple-negative PDX TN-1 cells were procured and transduced with dTomato^36^.

### Bioinformatics

Oncomine (www.oncomine.org) was used to mine human breast cancer microarray data comparing the expression of connexins including Cx26 in seven different TNBC (2,408 patient samples) datasets with normal breast tissue (250 samples). *Cx26* mRNA expression was assessed across multiple breast cancer subtypes using the breast cancer gene expression miner (version 4.0; http://bcgenex.centregauducheau.fr/). The diagnostic values of the respective genes were analyzed by KM plotter to obtain KM survival plots in which the number at risk is indicated below the main plot (http://kmplot.com/analysis/index.php?*p* = service&cancer = breast). Hazard ratio and 95% confidence intervals and log-rank *p* values were calculated and displayed on the webpage.

### Immunohistochemical staining

An institutional review board-approved protocol was obtained for the procurement of TNBC tissues for Cx26 staining. The staining patterns of Cx26 in non-tumor and tumor tissues of TNBC were assessed by a board certified pathologist.

### Fluorescence-activated cell sorting for CSC enrichment

To enrich for CSCs, MDA-MB-231 or HCC70 cells transduced for GFP NANOG promoter reporter were subjected to a BD FACS Aria II at a concentration of 1 million cells/mL and sorted according to GFP expression levels with MDA-MB-231 or HCC70 parental cells was used as a control to define negativity for GFP expression. Data analysis was performed using the FlowJo software (Tree Star Inc.).

The ALDEFLUOR kit (StemCell Technologies, Durham, NC, USA) was also used to enrich the CSC population based on the high ALDH enzymatic activity^35^. Cells freshly dissociated from the breast cancer PDX TN-1 xenografts were suspended in ALDEFLUOR assay buffer containing ALDH substrate and incubated for 30–60 min at 37 °C. As a negative control, an aliquot of each sample of cells was treated with diethylaminobenzaldehyde, a specific ALDH inhibitor. The sorting gates were established as described in the kit as per the manufacturer’s protocol, and cells were sorted into ALDH+ (CSCs) and ALDH− (non-CSCs) accordingly using BD FACS Aria II.

### Lentiviral production and infection

shRNAs and Cx26-transducing lentiviruses were prepared as described^66,67^. 293T cells were cultured in DMEM supplemented with 10% FCS. 293T cells were co-transfected with the packaging vectors psPAX2 and pMD2.G (Addgene, Cambridge, MA, USA) and lentiviral vectors directing expression of shRNA (Sigma, St. Louis, MO, USA) specific to *GJB2* (TRCN0000059893 (sh1), TRCN0000430109 (sh2), TRCN0000419197 (sh3), TRCN0000059894, TRCN000005895, TRCN0000005896, TRCN000005897, TRCN0000412781, TRCN0000422191), *NANOG* (TRCN0000004884 (sh1), TRCN0000004885 (sh2), TRCN0000004887, TRCN0000004888), *FAK* (TRCN0000121127, TRCN0000121209, TRCN0000121319, TRCN0000121207, TRCN0000001617, TRCN0000196310, TRCN0000121318, TRCN0000194984, TRCN0000121129, TRCN0000001620), a NT control shRNA (SHC002), and overexpression vector for *GJB2* (Cx26), *NANOG*, *FAK*, or an empty vector (Applied Biological Materials, Richmond, BC, Canada). Media of the 293T cultures were changed 18 h after transfection, and viral particles were harvested at 48 and 72 h, concentrated with polyethylene glycol precipitation, and stored at −80 °C for future use. Viral infections were carried out in MCF7 parental cells, MDA-MB-231/HCC70 parental cells, CSCs, and non-CSCs. Transduced cells were selected by their resistance to 2 μg/mL puromycin for 24–48 h. Following transduction, the cells were cultured in puromycin-free medium for 24 h before harvesting.

### Protein stability assay

MDA-MB-231 or HCC70 CSCs with or without *Cx26* silencing were treated with CHX at a concentration of 100 μg/mL immediately following 24-h puromycin selection. Non-target and shRNA-silenced cells were subsequently harvested at different times after CHX addition. Equal amounts of protein from each cell lysate were analyzed by probing for NANOG by immunoblotting. The half-life of Cx26 in MDA-MB-231 and HCC70 NANOG-GFP cells was determined by CHX chase assay. Flow-sorted MDA-MB-231 and HCC70 NANOG-GFP CSCs and non-CSCs were treated with CHX (100 μg/mL) for the indicated time, and western blotting was performed. The levels of Cx26 remaining at each time point were quantified as the percentage of initial Cx26 levels (0 h of CHX treatment).

### Immunoblotting

Cell lysates (20 μg total protein) as described^34^ were resolved by 10% sodium dodecyl sulfate-polyacrylamide gel electrophoresis and electrotransferred to PVDF membrane. After blocking, membranes were incubated overnight at 4  °C with primary antibodies against Cx26 (Invitrogen, Grand Island, NY, USA), Cx43 (Cell Signaling, Danvers, MA, USA), NANOG (Cell Signaling, Danvers, MA, USA), GFP (Invitrogen, Grand Island, NY, USA), SOX2 (Cell Signaling, Danvers, MA, USA), OCT4 (Cell Signaling, Danvers, MA, USA), phospho-FAK (Y397, Y576, Y925) (Cell Signaling, Danvers, MA, USA), total FAK (Cell Signaling, Danvers, MA, USA), and/or β-actin (Santa Cruz, Dallas, TX, USA), followed by incubation with secondary anti-mouse or anti-rabbit immunoglobulin G (IgG) antibodies conjugated to horseradish peroxidase (Thermo Scientific, Waltham, MA, USA). Immunoreactive bands were visualized by exposing films to luminescent signals generated after incubating the membrane with Pierce ECL plus (Thermo Scientific, Waltham, MA, USA). Copies of the uncropped blot scans are provided in the supplementary information (Supplementary Figs. 14–38) with associated molecular weights indicated for each antibody.

### Quantitative real-time PCR

Quantitative real-time PCR (qPCR) was performed using an ABI 7900HT system with SYBR-Green MasterMix (Qiagen, Valencia, CA, USA). Briefly, total RNA was extracted from cells using the RNeasy kit (Qiagen, Valencia, CA, USA), and complementary DNA was synthesized using the Superscript III kit (Invitrogen, Grand Island, NY, USA). For qPCR analysis, the threshold cycle (*C*_T_) values for each gene were normalized to expression levels of *β-actin*. Dissociation curves were evaluated for primer fidelity. The primers used are proved in Supplementary Table 2.

### Limiting dilution assays

For tumorsphere-formation assays, cells were cultured in serial dilutions in a 96-well plate for nonadherent culture (Sarstedt, Germany) per condition with 200 μl serum-free DMEM/F12 medium supplemented with 20 ng/mL basic fibroblast growth factor (Invitrogen, Grand Island, NY, USA), 10 ng/mL epidermal growth factor (BioSource, Grand Island, NY, USA), 2% B27 (vol/vol) (Invitrogen, Grand Island, NY, USA), and 10 μg/mL insulin (Sigma, St. Louis, MO, USA). Tumorsphere-formation was scored after 2 weeks under a phase contrast microscope. The frequency of sphere-forming cell was calculated accordingly using an extreme limiting dilution algorithm (ELDA) (http://bioinf.wehi.edu.au/software/elda/)^68^.

### In vivo tumor formation

Nonobese diabetic severe combined immunodeficiency gamma mice were purchased from the Biological Resource Unit at the Cleveland Clinic. All mice were maintained in micro-isolator units with free access to food and water. All mouse procedures were performed with adherence to protocols approved by the Institute Animal Care and Use Committee at the Lerner Research Institute of the Cleveland Clinic.

Cx26-silenced and NT control MDA-MB-231 or HCC70 CSCs were subcutaneously transplanted into the right flank of female mice at 6 weeks of age in serial dilutions of 8,000, 80,000, and 800,000 cells per injection. Injections of MDA-MB-231 or HCC70 non-CSCs overexpressing Cx26 or empty vector were also conducted as described above. Mice were monitored every day until the endpoint of day 40. Palpable tumors with a cross-sectional area >2 mm^2^ were taken as a positive read for tumor formation. The stem cell frequencies were calculated using an ELDA (http://bioinf.wehi.edu.au/software/elda/)^68^.

### Immunoprecipitation

Immunoprecipitation was performed by incubating the cell lysates with the indicated antibodies and the corresponding control non-immune IgG overnight at 4 °C. Protein A/G agarose beads (Santa Cruz, Dallas, TX, USA) were washed 3–4 times at 4 °C. The washed beads were incubated with the antibody/lysate mix for 2 h at 4 °C. The beads were then washed 3–4 times at 4 °C. Laemmli sample buffer was then added to the beads and boiled for 5 min to release precipitated proteins from the beads. The released proteins were analyzed by immunoblotting as described above.

### Transfection of Cx26 and pFAK mutant expression plasmids

MDA-MB-231 parental cells, NANOG-GFP and PDX TN-1 cells were transfected with the RFP-tagged or GFP-tagged WT or mutant Cx26 expression plasmids^52^ using X-tremeGENE HP DNA Transfection reagent (Roche, Indianapolis, IN, USA) according to the manufacturer’s protocol. The transfected cells were identified based on their RFP or GFP fluorescence. These cells were used for fluorescence microscopy to determine subcellular localization of fusion Cx26 protein, sphere-formation analysis, and also for immunoprecipitation for pull down using anti-GFP antibody.

MDA-MB-231 CSCs were transfected with HA-tagged FAK autophosphorylation mutant (pKH3-Y397F) or a WT pFAK control (pKH3 WT-FAK) using reagent’s name. Both constructs encoding full-length FAK^69,70^. Co-immunoprecipitation studies were performed using anti-HA antibody (Cell Signaling Technologies) in the transfected MDA-MB-231 CSCs.

### Immunofluorescence microscopy

To visualize the expression and localization of Cx26 in MDA-MB-231 and HCC70 parental cells and CSCs, the cells were plated on a coverslip placed in a 6-well plate. Cells were fixed with 4% paraformaldehyde for 15 min and washed three times with phosphate-buffered saline (PBS) containing 0.1% Triton X-100 for 5 min each. After washing, cells were permeabilized and blocked in 5% fetal bovine serum with 0.1% Triton X-100 in 1 × PBS for 1 h. Primary antibodies (Cx26, Lamin B1, and GM-130) and AF-647-labeled phalloidin were used to stain cells overnight at 4 °C. The following day, cells were washed three times with PBS for 5 min each, and the appropriate secondary antibody was applied for 1 h at room temperature. After secondary antibody incubation, cells were washed three times with PBS for 5 min each and counterstained with 4′,6-diamidino-2-phenylindole or Hoechst 33342 for 5 min. Afterwards, cells were washed three times with PBS for 5 min each. The coverslips were mounted using FluorSave Reagent (VWR International, Radnor, PA, USA). Cells were imaged using a confocal microscope, and images were prepared in using Adobe Photoshop. Single plane images and three-dimensional reconstructions of areas were generated in Imaris software.

### Subcellular fractionation

In order to separate a fraction of plasma membrane and a fraction of post-nuclear cytosol and organelles, cells were grown in 15 cm dishes to 90–100% confluence. Cells were then placed on ice, the media were aspirated, and cells were scraped in 3 mL of ice-cold buffer (250 mM sucrose, 1 mM EDTA, 20 mM tricine, pH 7.8). Cells were pelleted by centrifugation at 1,400 × *g* for 5 min. The pellet was resuspended in 1 mL of the buffer and homogenized using a Dounce-type homogenizer. The post-nuclear cytoplasmic fraction was removed and stored on ice. The pellet was resuspended in 1 mL of the buffer, homogenized, and centrifuged at 1,000 × *g* for 10 min. The two post-nuclear cytoplasmic fractions were combined and layered on top of 30% Percoll (Sigma, St. Louis, MO, USA) in the buffer and centrifuged at 84,000 × *g* for 30 min at 4 °C. The observed visible band of plasma membrane and the cytosol and organelle fraction were collected and diluted with the buffer. The plasma membrane fraction was centrifuged at 105,000 × *g* for 90 min to remove the Percoll^23^. The membrane fraction was resuspended in the buffer with protease and phosphatase inhibitors. The samples were then immunoblotted with the indicated antibodies.

To obtain nuclear and cytoplasmic fractions, we used the following procedure. Cytoplasmic extraction from pelleted cells were performed by resuspension in cytoplasmic extraction buffer (HEPES (10 mM, pH 7.9), NaCl (50 mM), sucrose (0.5 M), EDTA (0.1 mM), EGTA (0.1 mM), Triton X-100 (0.5%), DTT (1 mM)). The lysates were centrifuged at 20,000 × *g* for 5 min at 4°C. The supernatants were saved as the cytoplasmic fraction. The pellets were washed in PBS twice followed by extraction of the nucleus by using nuclear extraction buffer (HEPES (10 mM, pH 7.9), KCl (10 mM), EDTA (0.1 mM), EGTA (0.1 mM), DTT (1 mM)). The resuspended pellets were centrifuged at 20,000 × *g* for 10 min at 4 °C. The supernatants were saved as the nuclear fraction.

### GJ dye diffusion assay

To quantify GJ-mediated intercellular diffusion, we performed a microinjection-based analysis in MDA-MB-231 CSCs^31^. MDA-MB-231 CSCs were plated on coverslips at sub-confluent density the day before the assay. A single cell within a cell cluster was co-injected with biocytin-rhodamine and Cy5-labeled IgG. Immediately after microinjection, time-lapse video microscopy was used to capture phase contrast, red (for biocytin-rhodamine), and far-red (for Cy5 IgG) fluorescent images. The Cy5 IgG image defines the initially injected donor cells, and the red signal outside this donor cell is the dye diffused out of the donor cells into the neighboring cells when GJ is functional.

### Statistical analysis

All the experiments presented were run in triplicate, unless otherwise mentioned. The values reported in the results are mean values ± standard deviation. One-way analysis of variance was used to calculate the statistical significance, and the *p* values are detailed in the text and figure legends.

### Data availability

The authors declare that data supporting the findings of this study are available within the paper and its Supplementary Information files.

## Electronic supplementary material

Supplementary Information

Description of Additional Supplementary Files

Supplementary Movie 1
