# Supplementary material for: Cx26 drives self-renewal in triple-negative breast cancer via interaction with NANOG and focal adhesion kinase
Source: Nat Commun. 2018 Feb 8;9:578. doi: 10.1038/s41467-018-02938-1 (PMC5805730; doi:10.1038/s41467-018-02938-1)
Supplement: Supplementary file 1 — Supplementary Information [file 41467_2018_2938_MOESM1_ESM.pdf]

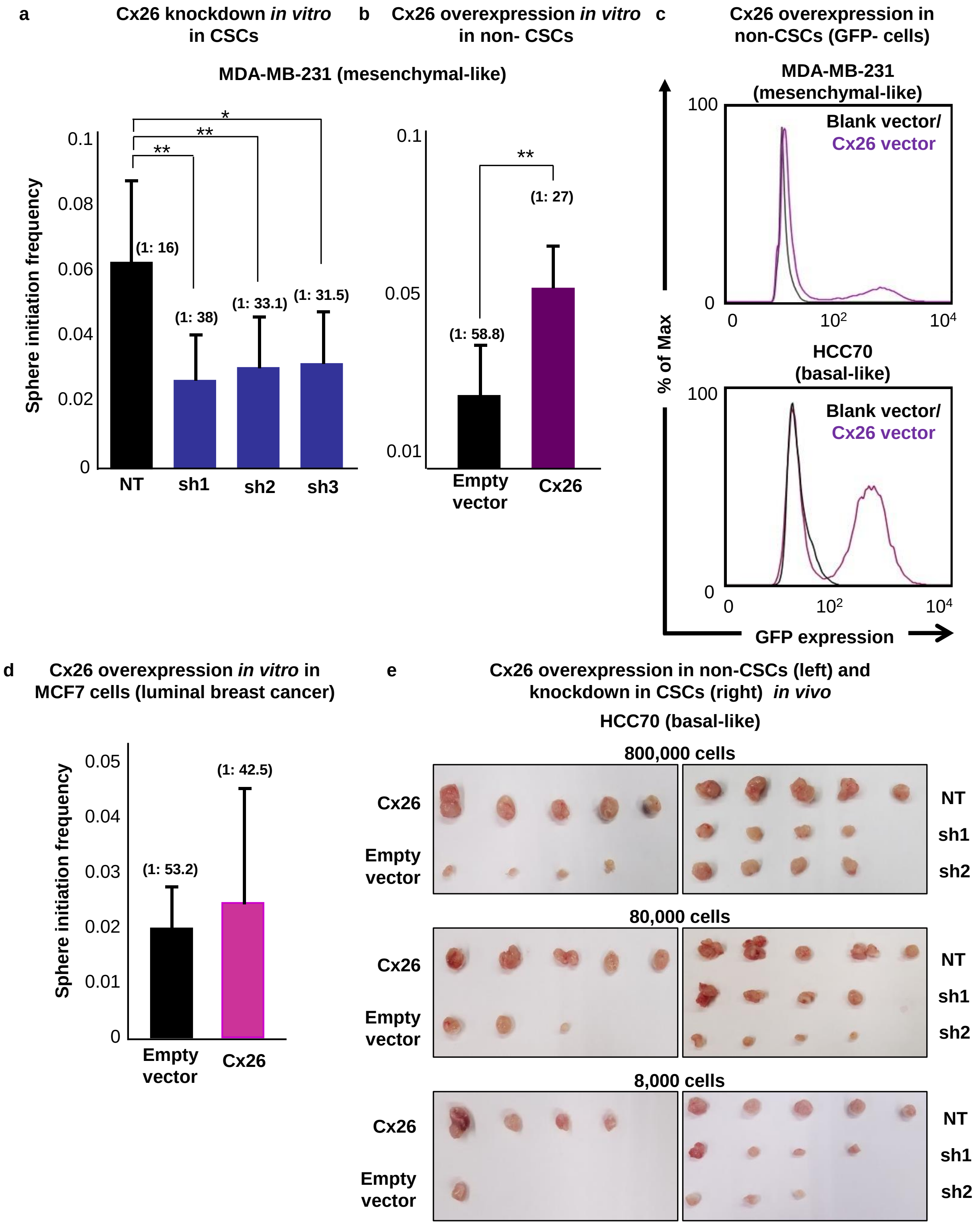
a
Cx26 knockdown in vitro in CSCs
b
Cx26 overexpression in vitro in non- CSCs
c
Cx26 overexpression in non-CSCs (GFP- cells)
MDA-MB-231 (mesenchymal-like)
Sphere initiation frequency
0.1
0.08
0.06
0.04
0.02
0
*
**
**
(1: 16)
(1: 38)
(1: 33.1)
(1: 31.5)
NT
sh1
sh2
sh3
0.1
0.05
0.01
**
(1: 27)
(1: 58.8)
Empty vector
Cx26
MDA-MB-231 (mesenchymal-like)
100
0
Blank vector/
Cx26 vector
0
$10^2$
$10^4$
% of Max
HCC70 (basal-like)
100
0
Blank vector/
Cx26 vector
0
$10^2$
$10^4$
GFP expression
d
Cx26 overexpression in vitro in MCF7 cells (luminal breast cancer)
Sphere initiation frequency
0.05
0.04
0.03
0.02
0.01
0
(1: 53.2)
(1: 42.5)
Empty vector
Cx26
e
Cx26 overexpression in non-CSCs (left) and knockdown in CSCs (right) in vivo
HCC70 (basal-like)
800,000 cells
Cx26
Empty vector
NT
sh1
sh2
80,000 cells
Cx26
Empty vector
NT
sh1
sh2
8,000 cells
Cx26
Empty vector
NT
sh1
sh2

**Supplementary Figure 1. Cx26 overexpression in non-TNBC and TNBC cell lines.** (**a, b**) Sphere-initiating frequencies in MDA-MB-231 CSCs transduced with non-targeting (NT) shRNA or Cx26-silencing shRNA constructs and MDA-MB-231 non-CSCs transduced with Cx26 overexpression vector or empty vector were determined by limiting dilution sphere-formation assays. The graphs compare the estimates of the stem cell frequencies of each group. (**c**) Overexpression of Cx26 in MDA-MB-231 and HCC70 non-CSCs induced the NANOG promoter GFP reporter as measured by fluorescence-activated cell sorting (FACS). GFP expression was analyzed using FlowJo software (Version 10). The black and purple histogram lines represent blank vector and Cx26 overexpression, respectively. (**d**) The stem cell frequency was not affected by the introduction of Cx26 overexpression vector into MCF7 cells compared with empty vector control as calculated by limiting dilution sphere-formation assays. (**e**) *In vivo* limiting dilution assays show decreased tumor initiation by Cx26-silenced HCC70 CSCs and increased tumor initiation in Cx26-overexpressed HCC70 non-CSCs compared with the control groups. (* $p < 0.05$, ** $p < 0.01$). All error bars indicate the upper and lower range.

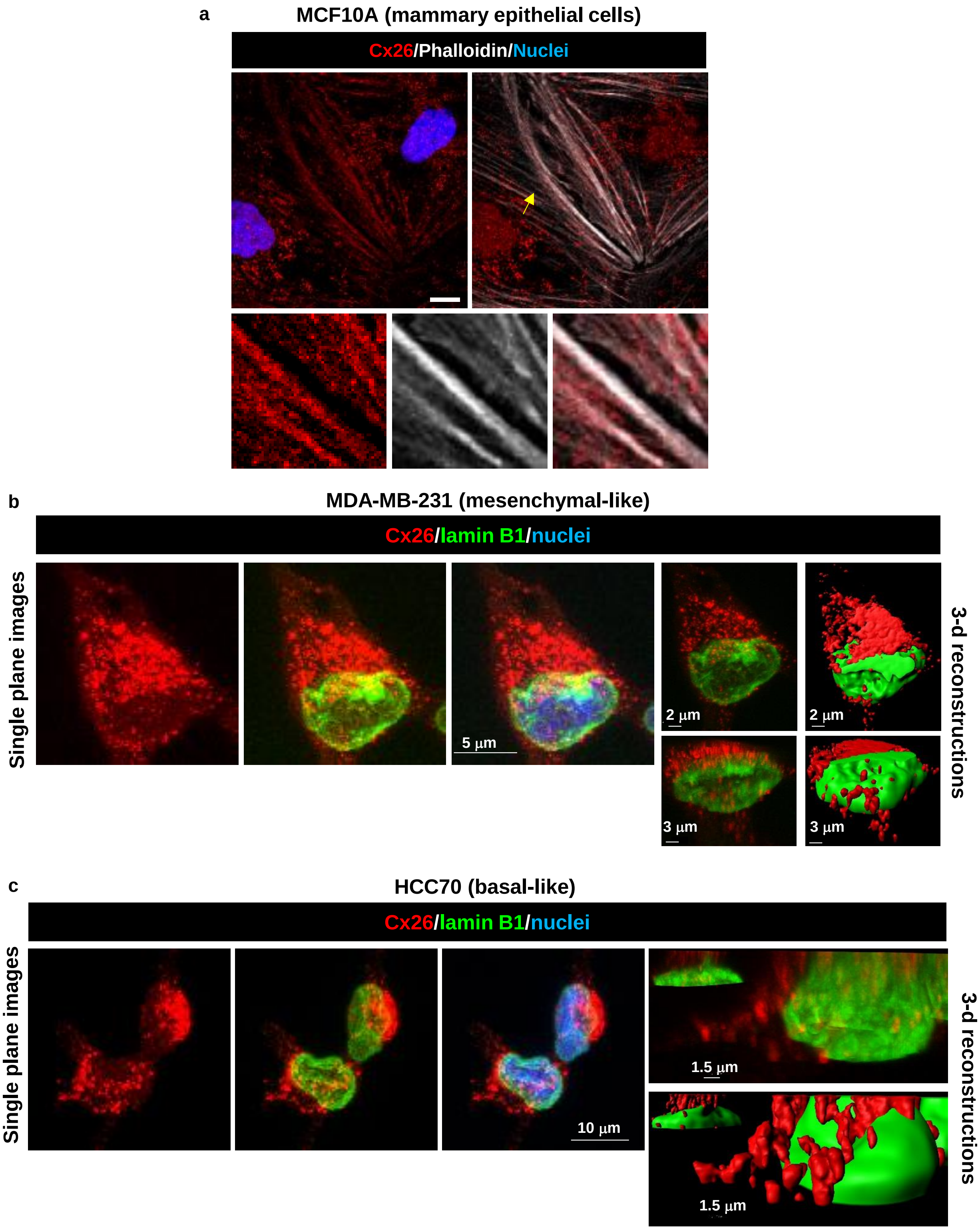
a
MCF10A (mammary epithelial cells)
Cx26/Phalloidin/Nuclei
b
MDA-MB-231 (mesenchymal-like)
Cx26/lamin B1/nuclei
Single plane images
5 µm
2 µm
2 µm
3 µm
3 µm
3-d reconstructions
c
HCC70 (basal-like)
Cx26/lamin B1/nuclei
Single plane images
10 µm
1.5 µm
1.5 µm
3-d reconstructions

**Supplementary Figure 2. Cx26 protein localization in TNBC (MDA-MB-231 and HCC70) and MCF10A cells.** (**a**) Representative confocal images of Cx26 staining (red) and phalloidin (white) in MCF10A cells. Nuclei are stained with DAPI (blue); yellow arrow indicates cell junctions. Scale bar represents 10 μm. (**b, c**) Representative single-plane confocal microscopy images and 3-D reconstructions of Cx26 staining (red) and Lamin B1 (green) in MDA-MB-231 and HCC70 cells. Nuclei were stained with Hoechst 33342. Scale bars for MDA-MB-231 cells represent 5 μm for single plane images, 2 μm for the top reconstructions and 3 μm for the bottom reconstructions. For HCC70 cells, scale bars represent 10 μm for single plane images and 1.5 μm for the reconstructions.

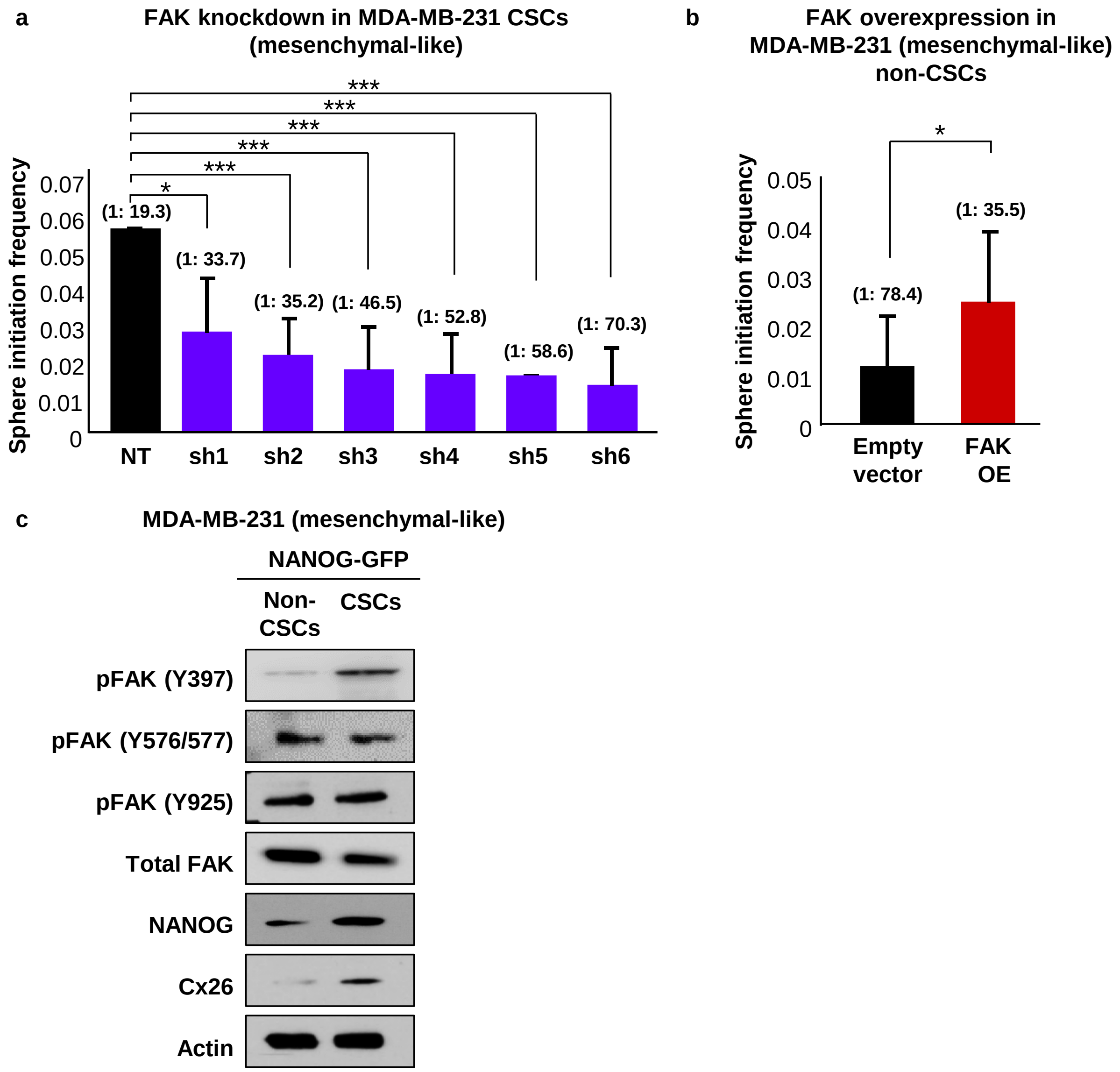


**Supplementary Figure 3. FAK is necessary and sufficient for CSC maintenance.** (**a**) Limiting dilution analysis graph shows sphere-forming abilities of NT control and FAK sh1-sh6-transduced MDA-MB-231 CSCs. (**b**) Limiting dilution analysis graph shows empty vector compared with the overexpression of FAK in MDA-MB-231 non-CSCs. (**c**) Lysates from sorted NANOG GFP-high (CSCs) and GFP-low (non-CSCs) MDA-MB-231 cells were probed for different tyrosine phosphorylation sites (pFAK) of FAK including Y576/577, Y925, and the autophosphorylation site Y397, total FAK, NANOG, and Cx26. Actin was used as a loading control. (* $p < 0.05$, *** $p < 0.001$). All error bars indicate the upper and lower range.

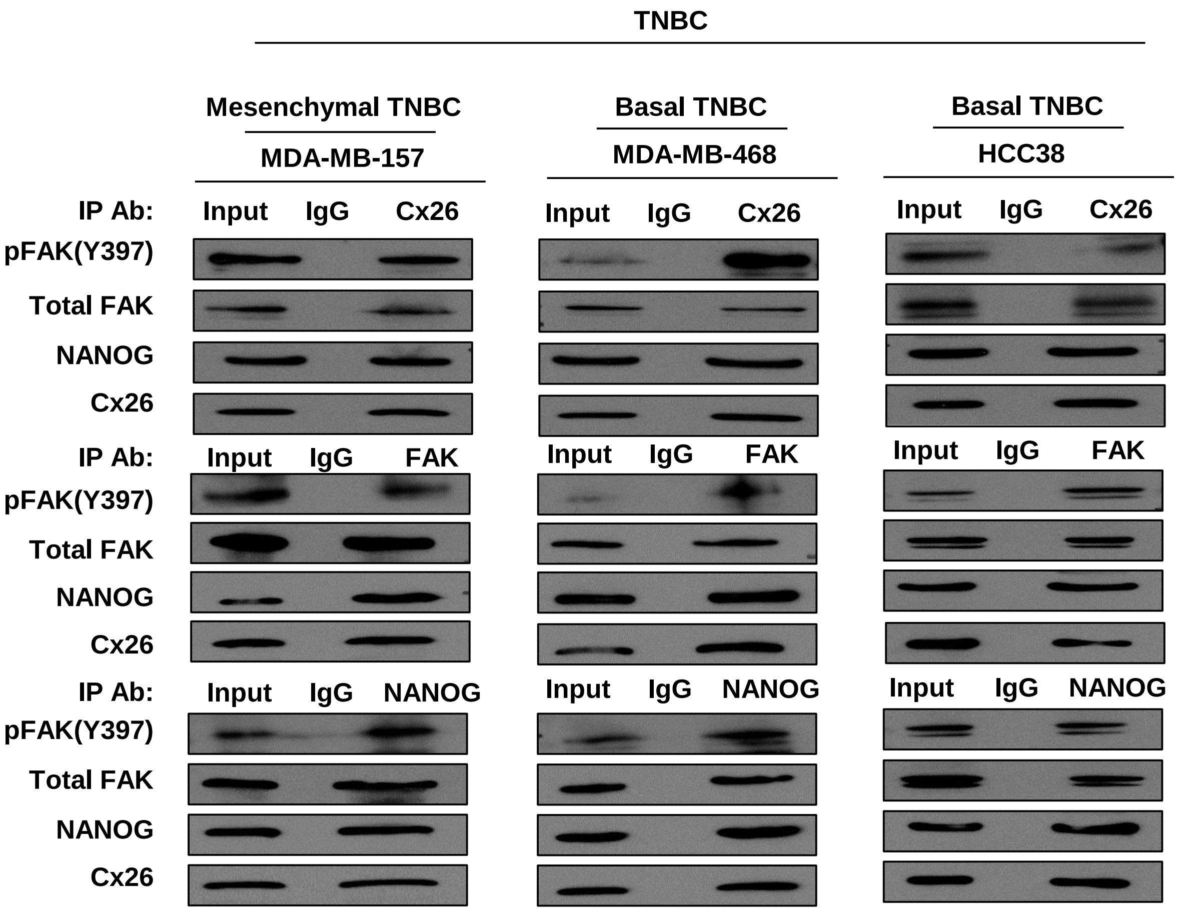


**Supplementary Figure 4. Cx26 forms a TNBC-specific complex with focal adhesion kinase (FAK) and NANOG.** Cell lysates from bulk cell cultures of mesenchymal-like TNBC (MDA-MB-157) and basal-like TNBC (MDA-MB-468, HCC38) were subjected to immunoprecipitation with anti-Cx26, anti-FAK, and anti-NANOG antibodies. pFAK (Y397), FAK, Cx26, and NANOG proteins in the precipitated complex were detected by western blotting using specific antibodies. Fifteen percent of the lysate used for immunoprecipitation was loaded as the input control. As a negative control, immunoprecipitation with the corresponding non-immune IgG was performed.

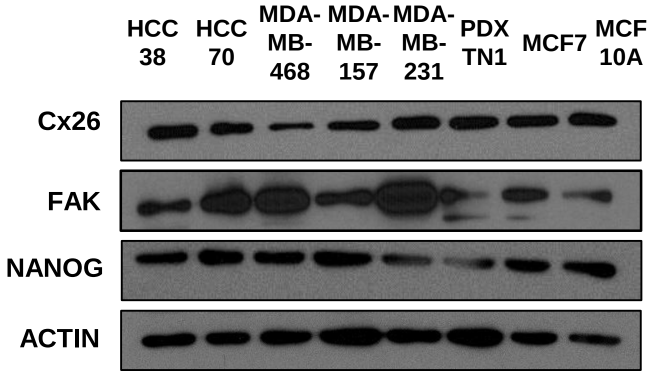


**Supplementary Figure 5. Cx26, NANOG, and FAK protein expression across different breast cancer subtypes.** Basal-like TNBC cell lines (HCC38, HCC70, MDA-MB-468), mesenchymal-like TNBC cell lines (MDA-MB-157, MDA-MB-231), luminal breast cancer (MCF7) cell line, and mammary epithelial cells (MCF10A) were probed for Cx26, NANOG, and FAK protein expression using the corresponding antibodies. Actin was used as a loading control.

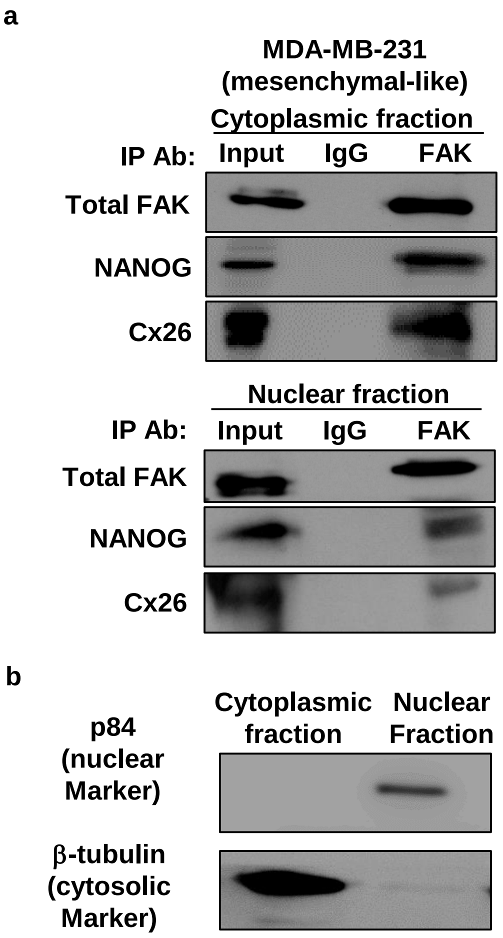


**Supplementary Figure 6. Nuclear and cytoplasmic localization of the Cx26/FAK/NANOG complex in MDA-MB-231 cells.** (**a**) The Cx26/NANOG/FAK ternary complex was detected in the cytoplasmic and the nuclear fractions of MDA-MB-231 cells by pull-down experiments using an antibody against FAK and probed for total FAK, NANOG, and Cx26. (**b**) Validation of nuclear and cytoplasmic fractionation was demonstrated by immunoblotting after fractionation with antibodies against p84 and β-tubulin to mark the nuclear and cytoplasmic compartments, respectively.

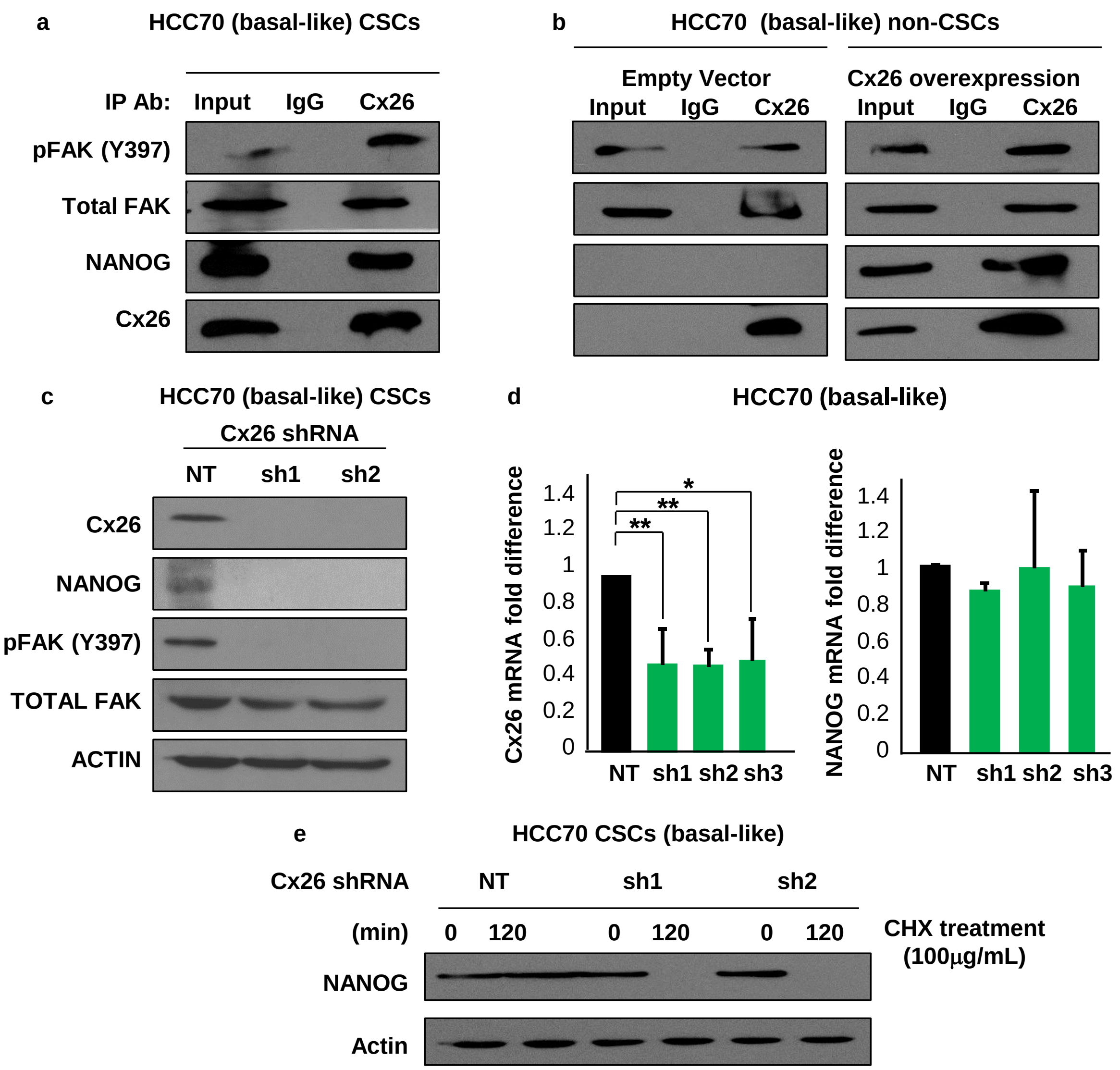


**Supplementary Figure 7. The Cx26/NANOG/FAK interaction is enriched in HCC70 CSCs, and Cx26 regulates NANOG stability.** (**a**) Immunoprecipitations using Cx26 antibody were performed in HCC70 CSCs and (**b**) non-CSCs transduced with either empty vector or Cx26-overexpression vector. The precipitates were probed for pFAK (Y397), total FAK, NANOG, and Cx26 by immunoblotting. (**c**) Immunoblots of the cell lysates from HCC70 NANOG-GFP CSCs that were silenced for Cx26 using two shRNA constructs each (sh1 and sh2) and from CSCs that received a non-targeting (NT) control shRNA were probed with NANOG, Cx26, pFAK (Y397), and total FAK antibodies. Actin was used as a loading control. (**d**) Fold difference in mRNA expression

of *Cx26* and *NANOG* in Cx26-silenced HCC70 CSCs compared with NT control was determined by qPCR. Actin was used as a normalization control. (* $p < 0.05$, ** $p < 0.01$). Error bars indicate standard deviation. (**e**) Cx26-silenced and non-targeting control (NT) HCC70 CSCs were treated with cycloheximide (CHX) at 0 and 120 minutes and probed for NANOG expression by immunoblotting. Actin was used as a loading control.

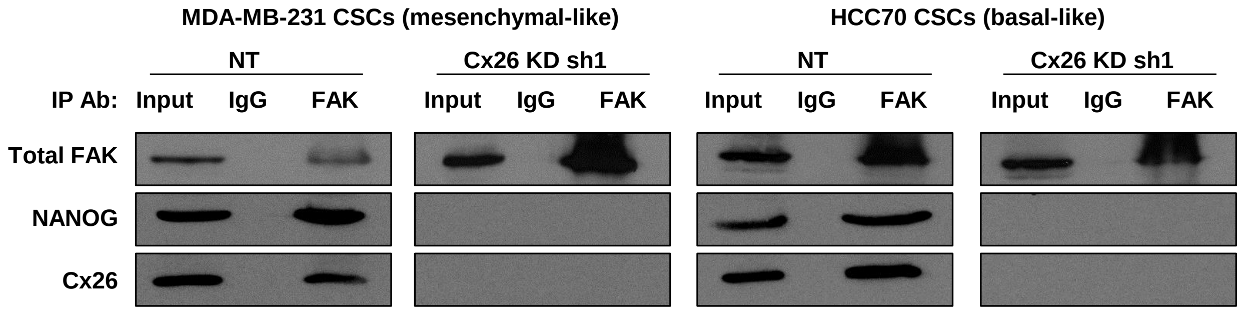


**Supplementary Figure 8. Cx26 knockdown disrupts Cx26/FAK/NANOG complex formation in MDA-MB-231 and HCC70 CSCs.** Lysates from Cx26-silenced and control MDA-MB-231 CSCs and HCC70 CSCs were immunoprecipitated using FAK antibody. The precipitates were probed with total FAK, NANOG, and Cx26 antibodies.

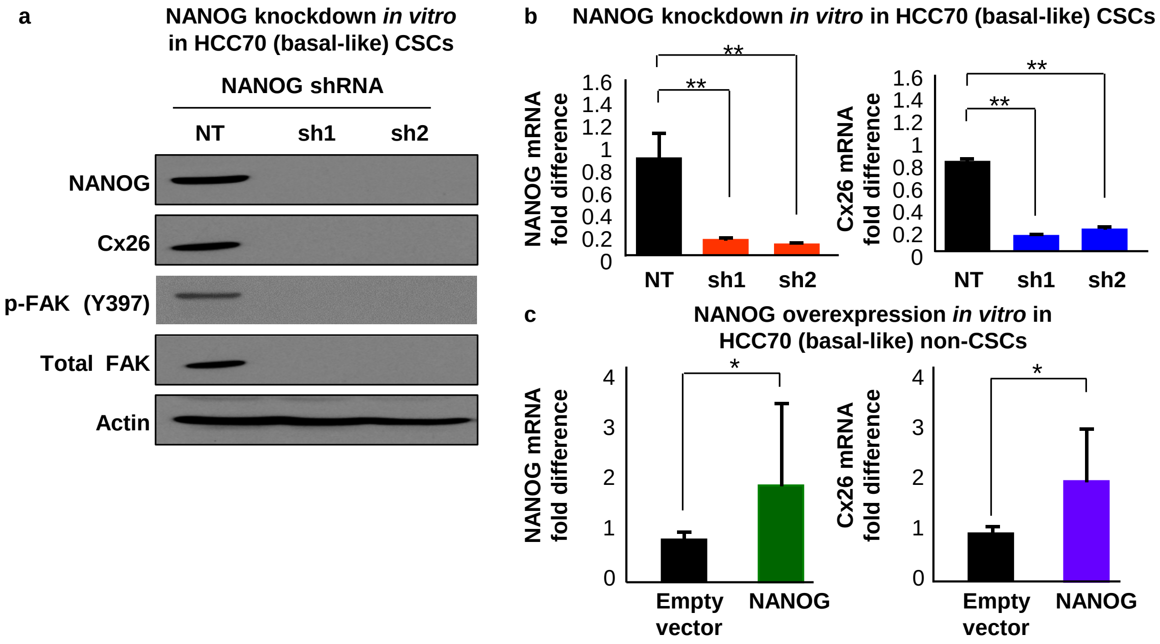


**Supplementary Figure 9. NANOG regulates Cx26 expression in HCC70 CSCs.** (**a**) Immunoblots of lysates from HCC70 NANOG-GFP CSCs silenced for NANOG using two shRNA constructs each and CSCs transduced with a non-targeting control were probed with Cx26, NANOG, pFAK (Y397), and total FAK antibodies. Actin was used as a loading control. (**b, c**) Fold difference in mRNA expression of *Cx26* and *NANOG* in NANOG-silenced HCC70 CSCs and NANOG-overexpressed HCC70 non-CSCs compared with their corresponding controls was determined by qPCR. Actin was used as a normalization control. (* $p < 0.05$, ** $p < 0.01$). Error bars indicate standard deviation.

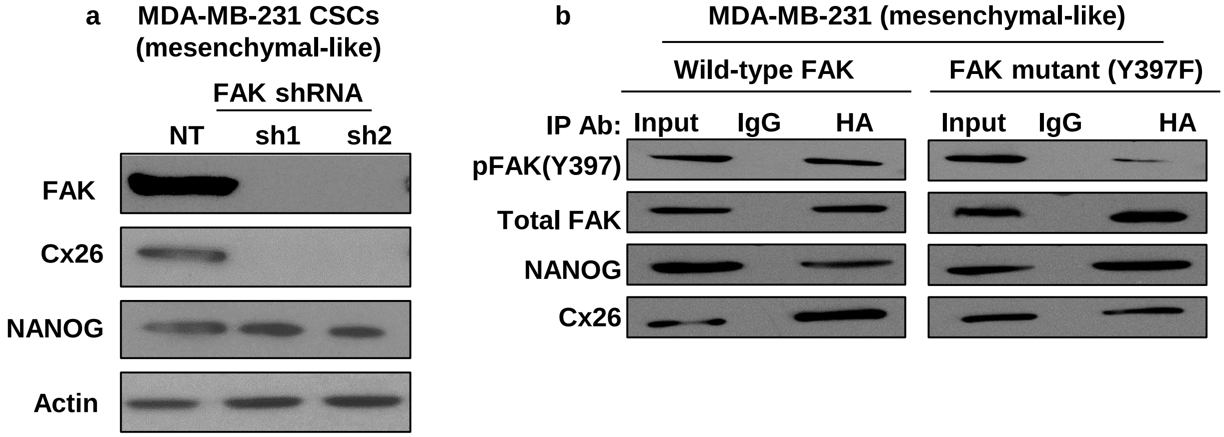


**Supplementary Figure 10. FAK regulates Cx26 expression.** (**a**) Immunoblots of the cell lysates from MDA-MB-231 NANOG-GFP CSCs silenced for FAK using two shRNA constructs each (sh1 and sh2) and from CSCs transduced with a non-targeting (NT) control were probed with NANOG, Cx26, and total FAK antibodies. Actin was used as a loading control. (**b**) Immunoprecipitation of MDA-MB-231 CSCs containing HA-tagged wild-type FAK or the pFAK mutant Y397F was performed using anti-HA (hemagglutinin) antibody. The precipitates were probed with pFAK (Y397), total FAK, NANOG, and Cx26 antibodies.

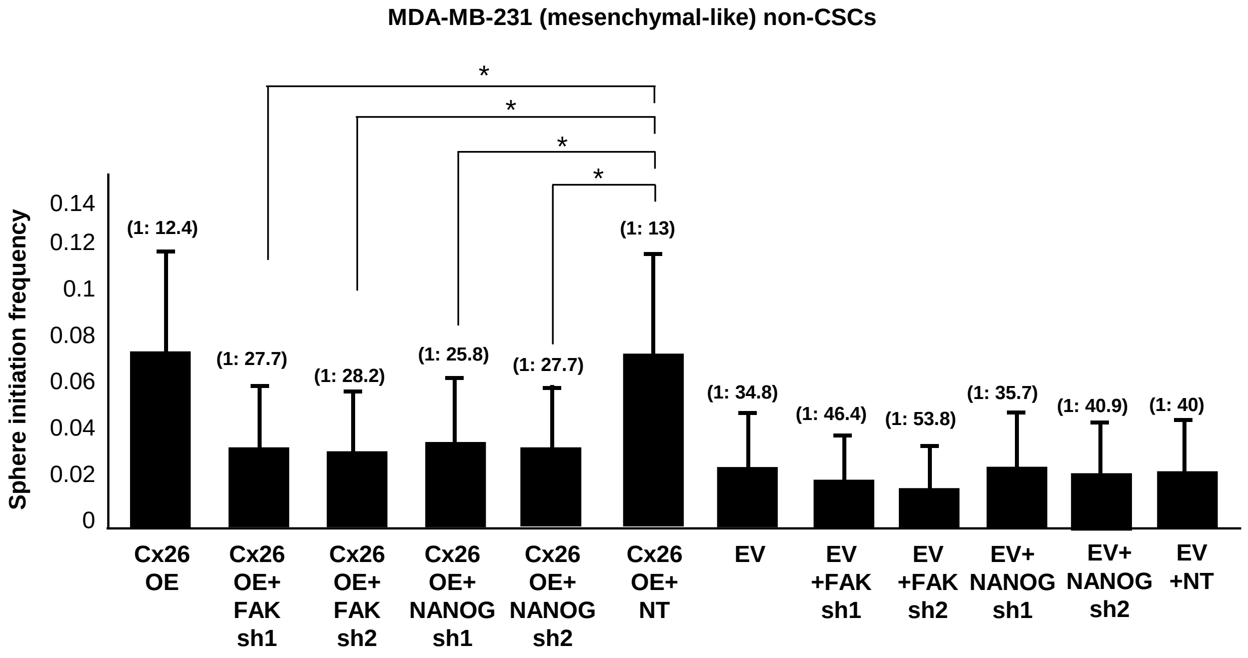


**Supplementary Figure 11. FAK or NANOG knockdown in Cx26-overexpressed MDA-MB-231 non-CSCs decreases sphere initiation frequency.** Stem cell frequencies in Cx26-overexpressed MDA-MB-231 non-CSCs containing FAK or NANOG knockdown compared with NT and/or empty vector controls were determined by limiting dilution sphere forming assays. (* $p < 0.05$). All error bars indicate the upper and lower range.

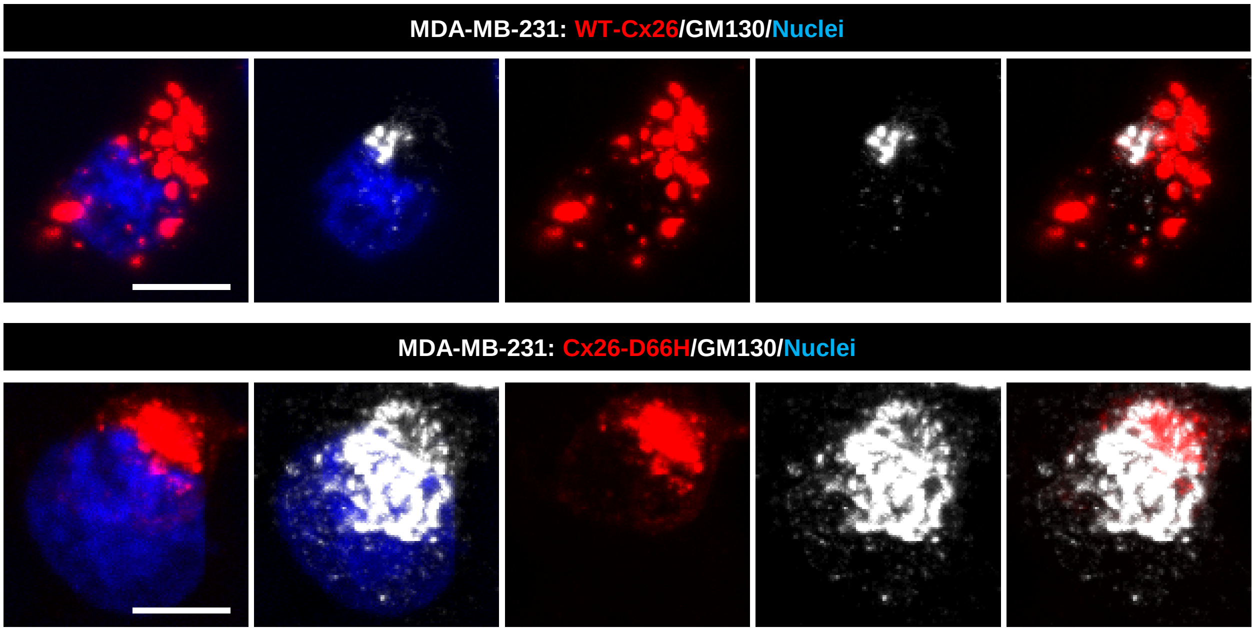


**Supplementary Figure 12. Mislocalization of the D66H Cx26 mutant in MDA-MB-231 parental cells.** The top panel shows immunofluorescence staining of RFP-tagged wild-type Cx26 and the Golgi marker GM130 in MDA-MB-231 parental cells. The bottom panel shows the RFP-tagged D66H Cx26 mutant and the Golgi marker GM130 in MDA-MB-231 parental cells. Scale bars represent 10 μm.

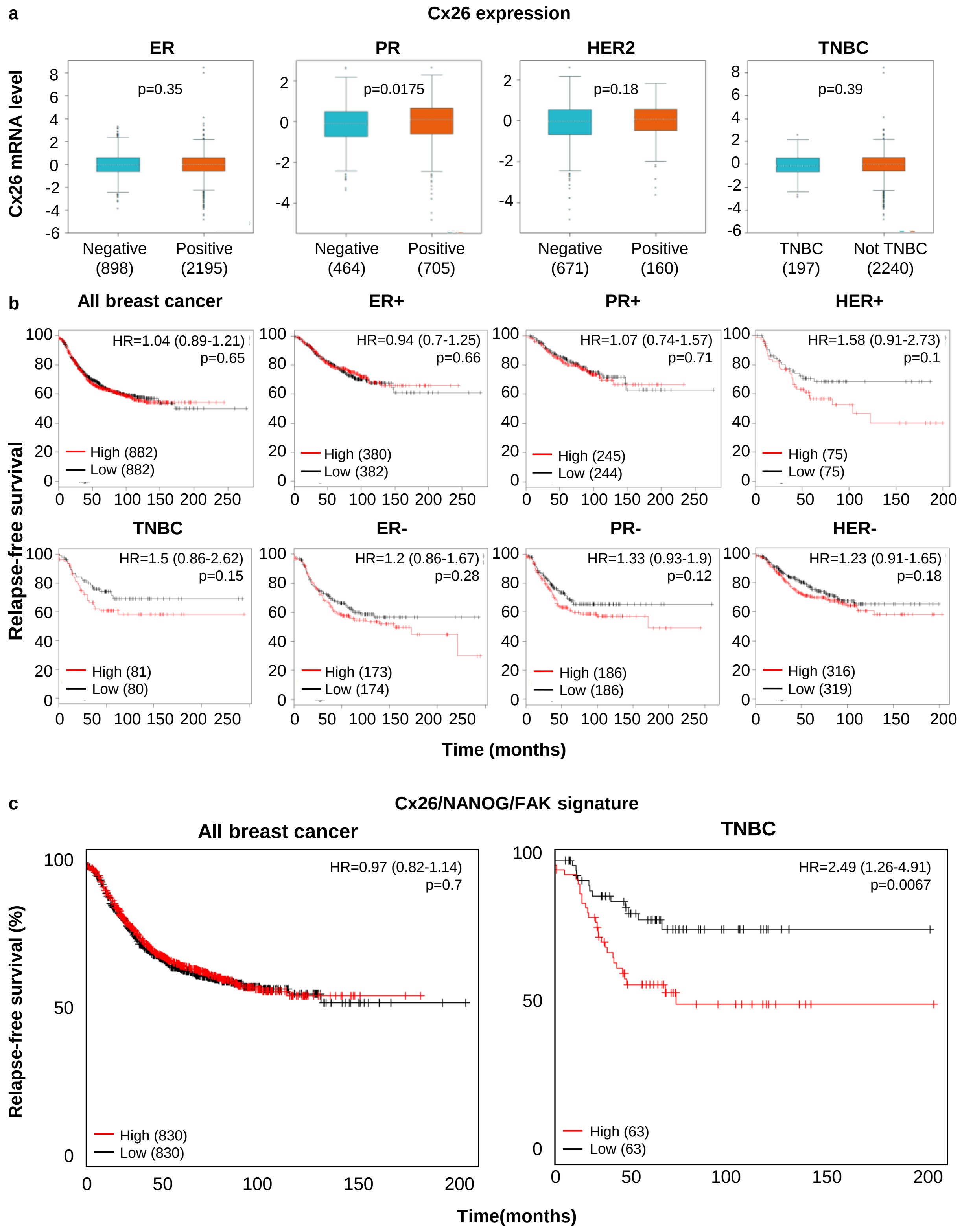
a
Cx26 expression
ER
PR
HER2
TNBC
Cx26 mRNA level
p=0.35
p=0.0175
p=0.18
p=0.39
Negative (898)
Positive (2195)
Negative (464)
Positive (705)
Negative (671)
Positive (160)
TNBC (197)
Not TNBC (2240)
b
All breast cancer
ER+
PR+
HER+
HR=1.04 (0.89-1.21) p=0.65
HR=0.94 (0.7-1.25) p=0.66
HR=1.07 (0.74-1.57) p=0.71
HR=1.58 (0.91-2.73) p=0.1
High (882) Low (882)
High (380) Low (382)
High (245) Low (244)
High (75) Low (75)
Relapse-free survival
TNBC
ER-
PR-
HER-
HR=1.5 (0.86-2.62) p=0.15
HR=1.2 (0.86-1.67) p=0.28
HR=1.33 (0.93-1.9) p=0.12
HR=1.23 (0.91-1.65) p=0.18
High (81) Low (80)
High (173) Low (174)
High (186) Low (186)
High (316) Low (319)
Time (months)
c
Cx26/NANOG/FAK signature
All breast cancer
TNBC
Relapse-free survival (%)
HR=0.97 (0.82-1.14) p=0.7
High (830) Low (830)
HR=2.49 (1.26-4.91) p=0.0067
High (63) Low (63)
Time(months)

**Supplementary Figure 13. Cx26 expression across breast cancer subtypes.** (**a**) Comparison of *Cx26* mRNA levels between the two cohorts of breast cancer patients based on different parameters; positive vs negative for estrogen receptor (ER), progesterone receptor (PR), and human epidermal growth factor receptor 2 (HER) and triple-negative (TNBC) vs. non-triple negative breast cancer. Number of patients per group and p-values are listed on plots. Data accessed from the breast cancer gene expression miner (version 4.0). (**b**) Kaplan-Meier plots demonstrate the correlation between Cx26 expression and relapse-free survival across different subtypes of breast cancer. Red lines represent patients above and black lines represent patients below the median *Cx26* mRNA levels. Number of patients per group is as follows: all breast cancer (882 in each group), ER+ (382 in low and 380 in high), PR+ (244 in low and 245 in high), HER2+ (75 in each group), TNBC (80 in low and 81 in high), ER- (174 in low and 173 in high), PR- (186 in each group), HER2- (319 in low and 316 in high). Hazard ratios (HR) per group and p-values are listed on the plots. Data was accessed from the KM-plotter. (**c**) Kaplan-Meier plots demonstrate the correlation between elevated expression of all the three genes (Cx26/FAK/NANOG) and relapse-free survival in the patients of all breast cancer types or the patients with TNBC.

Figure 1b

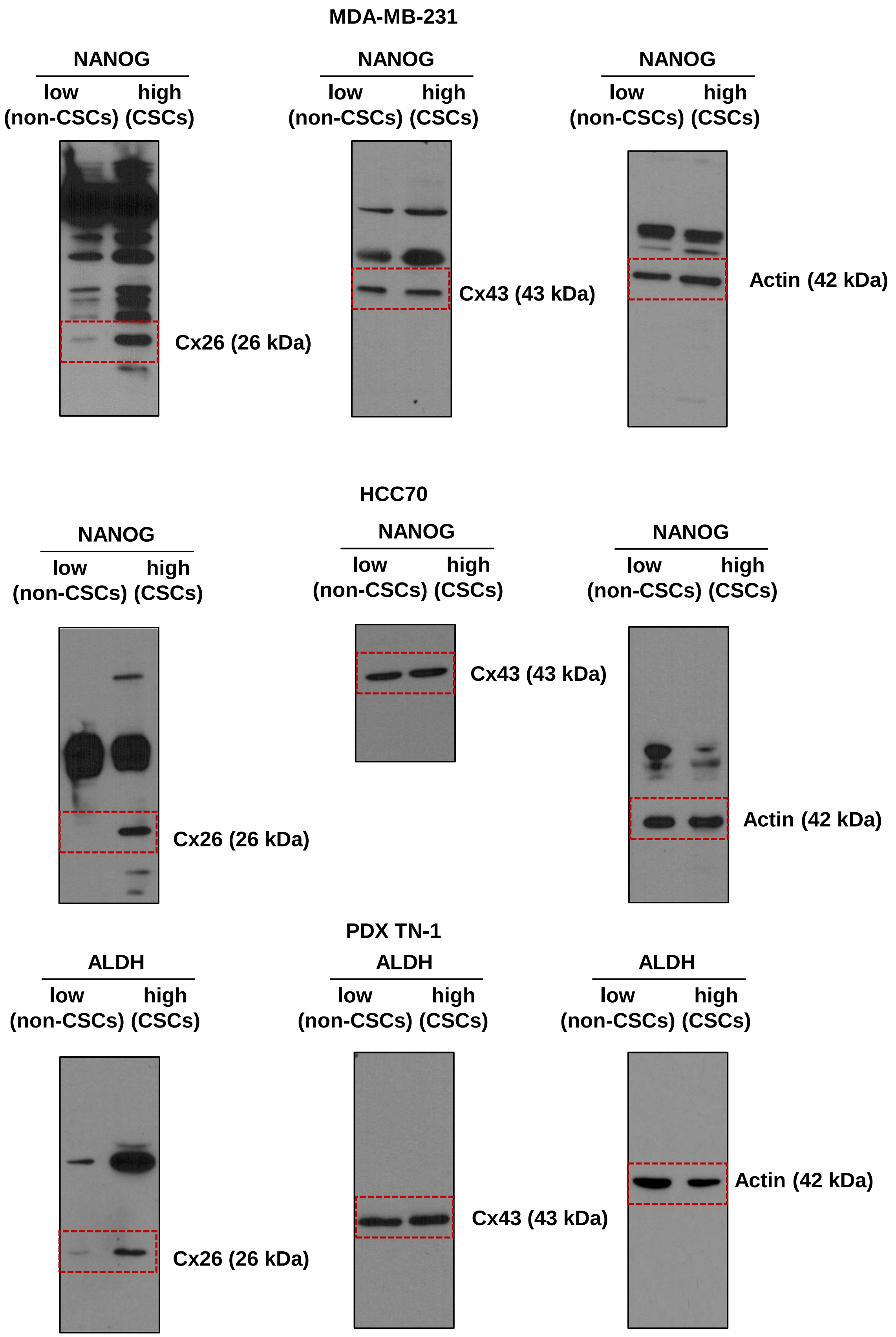
MDA-MB-231
NANOG
low
high
(non-CSCs) (CSCs)
Cx26 (26 kDa)
Cx43 (43 kDa)
Actin (42 kDa)
HCC70
NANOG
low
high
(non-CSCs) (CSCs)
Cx26 (26 kDa)
Cx43 (43 kDa)
Actin (42 kDa)
PDX TN-1
ALDH
low
high
(non-CSCs) (CSCs)
Cx26 (26 kDa)
Cx43 (43 kDa)
Actin (42 kDa)

**Supplementary Figure 14. Original uncropped gels for Figure 1b.** Original gels for the immunoblots presented in Figure 1. Area cropped in indicated with red box, individual molecular weights of each individual antibody indicated on immunoblot.

Figure 2a

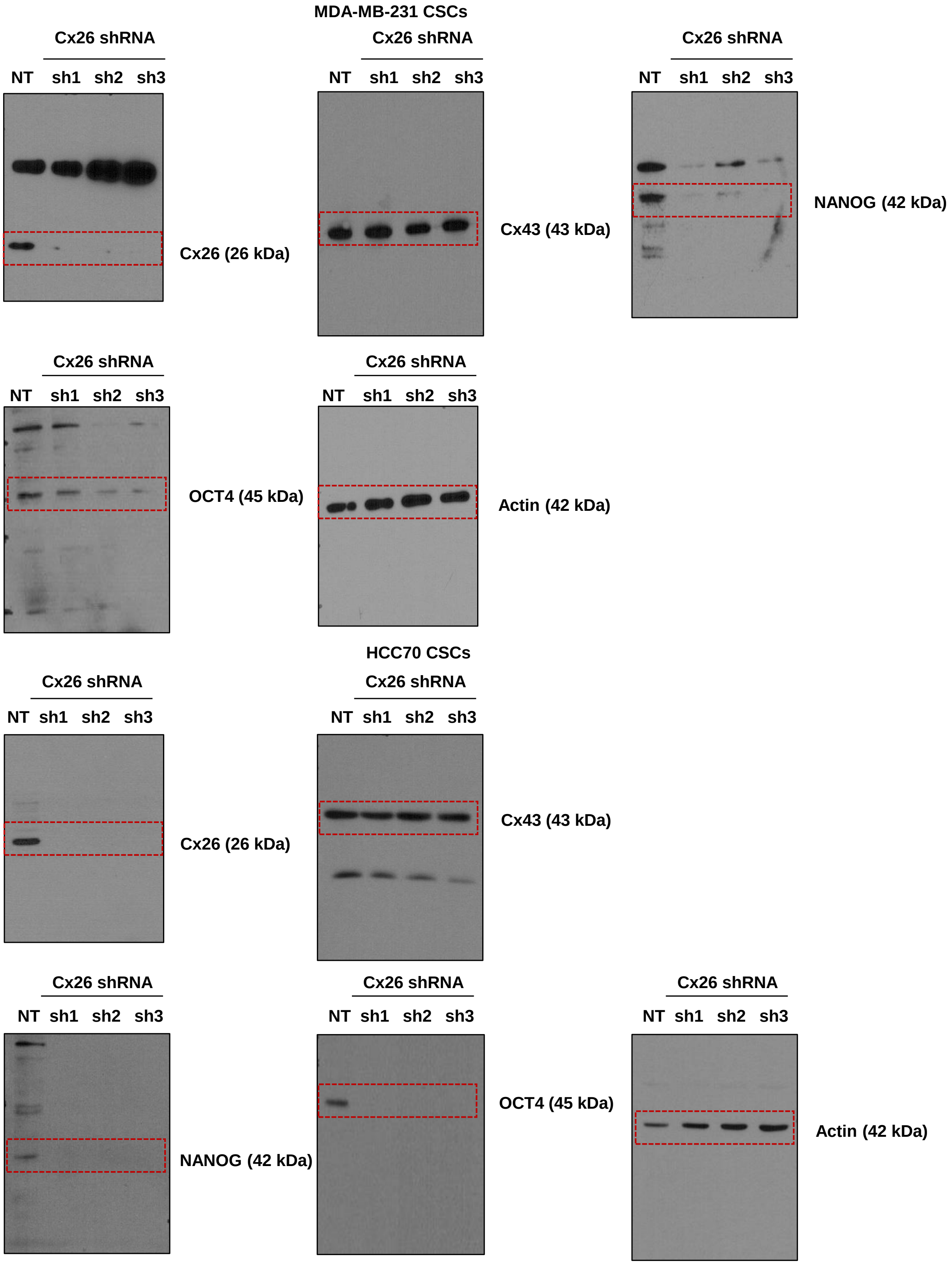
MDA-MB-231 CSCs
Cx26 shRNA
NT sh1 sh2 sh3
Cx26 (26 kDa)
Cx43 (43 kDa)
NANOG (42 kDa)
OCT4 (45 kDa)
Actin (42 kDa)
HCC70 CSCs
Cx26 shRNA
NT sh1 sh2 sh3
Cx26 (26 kDa)
Cx43 (43 kDa)
NANOG (42 kDa)
OCT4 (45 kDa)
Actin (42 kDa)

**Supplementary Figure 15. Original uncropped gels for Figure 2a.** Original gels for the immunoblots presented in Figure 2A. Area cropped in indicated with red box, individual molecular weights of each individual antibody indicated on immunoblot.

Figure 2c

MDA-MB-231 non-CSCs

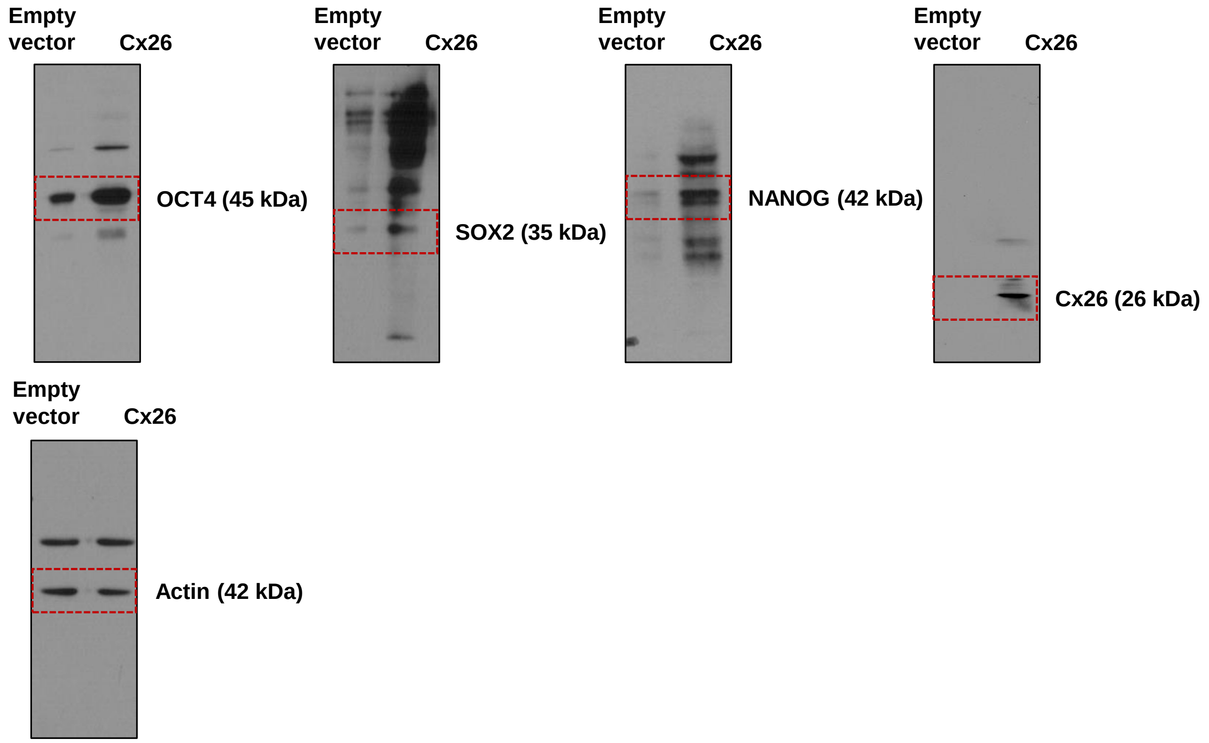


HCC70 non-CSCs

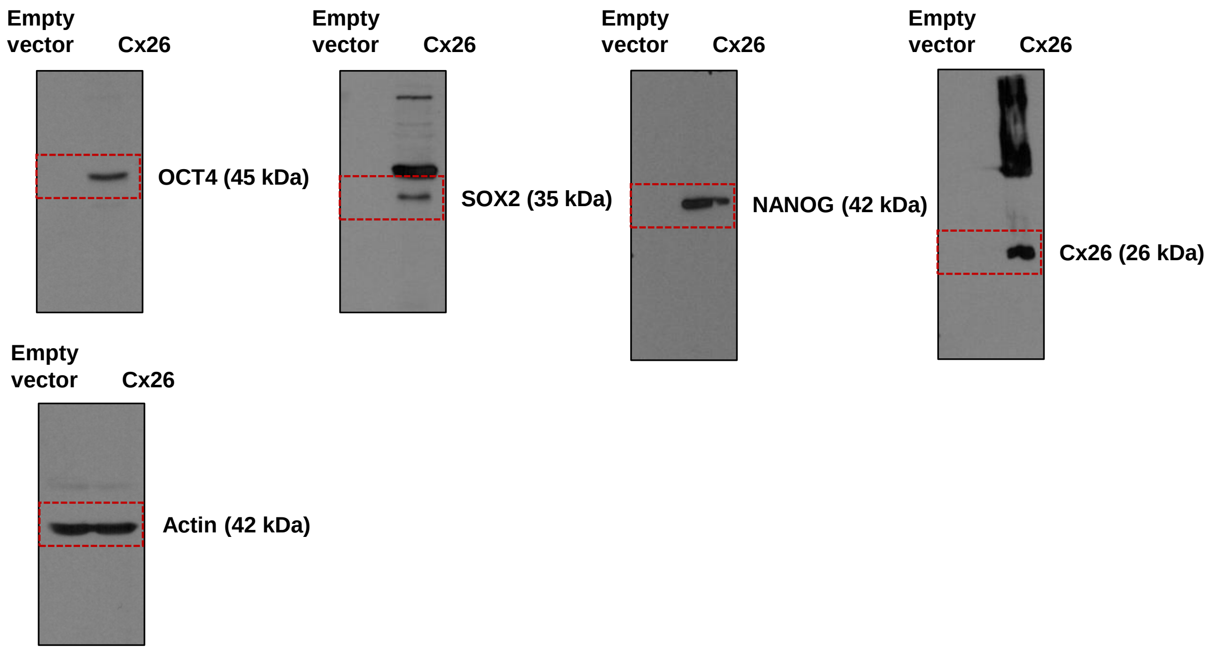

**Supplementary Figure 16. Original uncropped gels for Figure 2c.** Original gels for the immunoblots presented in Figure 2C. Area cropped in indicated with red box, individual molecular weights of each individual antibody indicated on immunoblot.

**Figure 3a**

**MDA-MB-231**

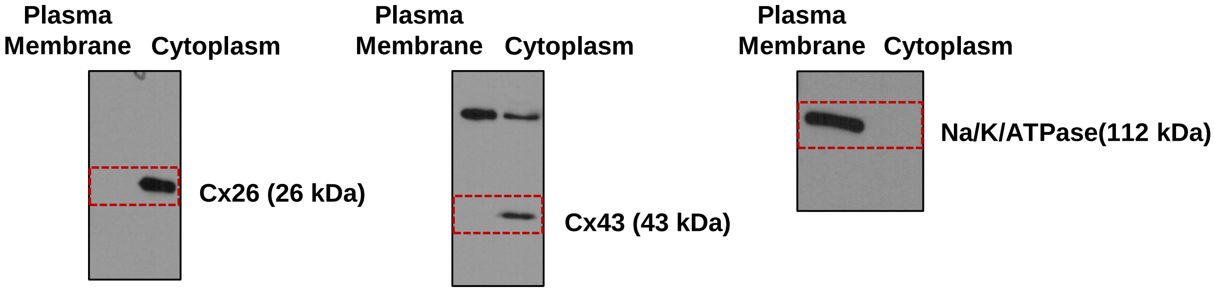


**Figure 3b**

**MCF7**

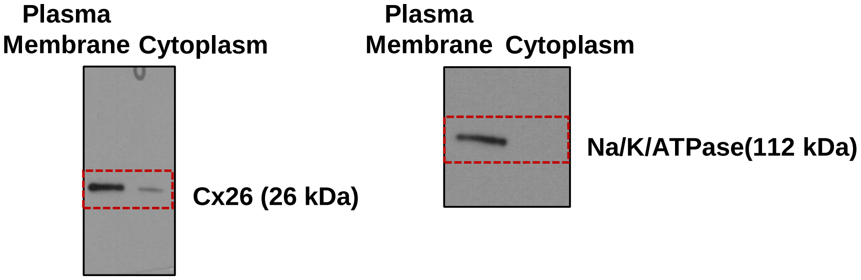


**Figure 3c**

**MCF10A**

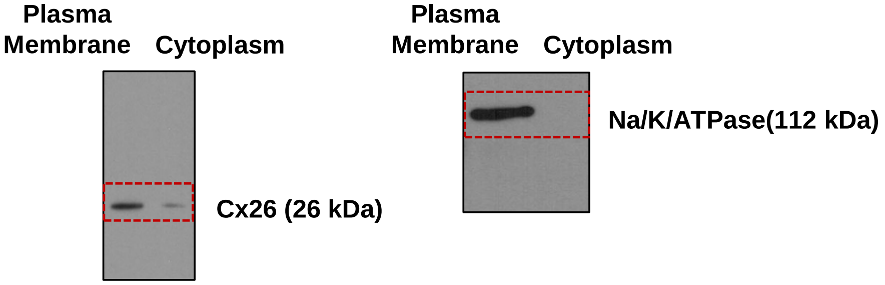


**Supplementary Figure 17. Original uncropped gels for Figure 3.** Original gels for the immunoblots presented in Figure 3. Area cropped in indicated with red box, individual molecular weights of each individual antibody indicated on immunoblot.

Figure 4a – MDA-MB-231 cells

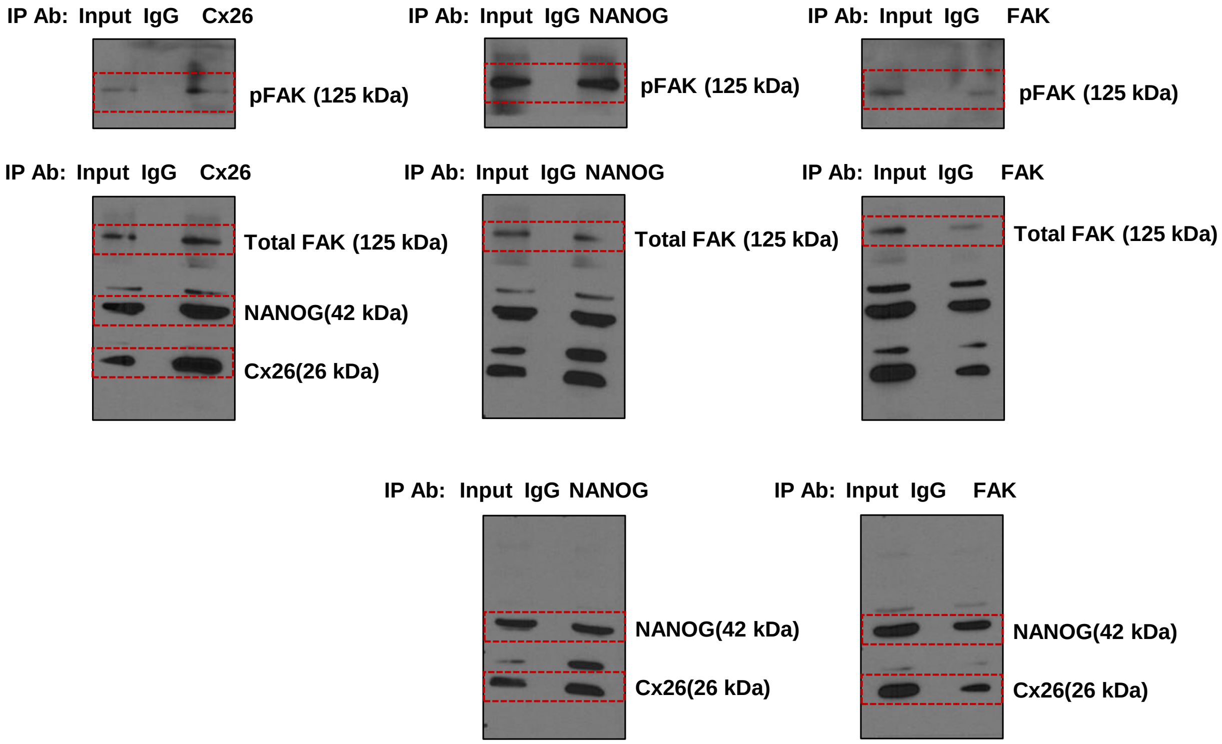


**Supplementary Figure 18. Original uncropped gels for MDA-MB-231 cells in Figure 4a.** Original gels for the immunoblots presented in Figure 4a with MDA-MB-231 cells. Area cropped in indicated with red box, individual molecular weights of each individual antibody indicated on immunoblot.

Figure 4a – HCC70 cells

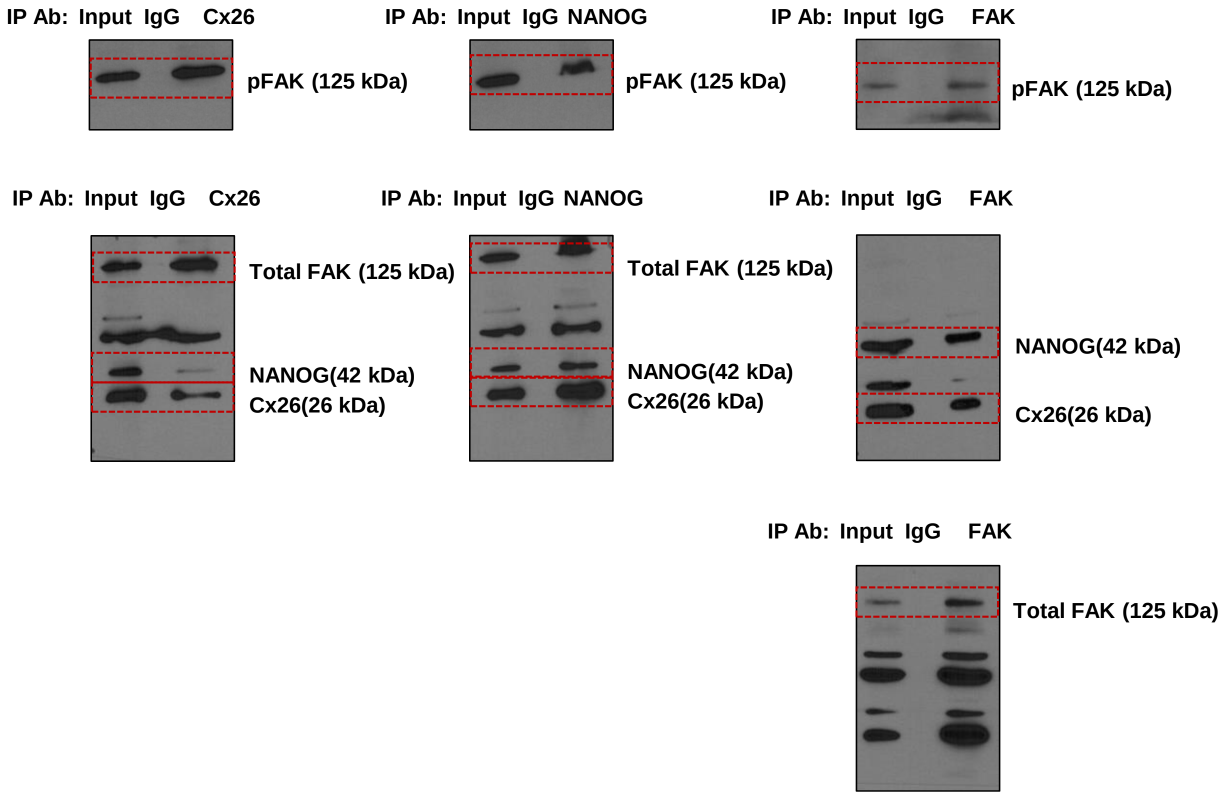


**Supplementary Figure 19. Original uncropped gels for HCC70 cells in Figure 4a.** Original gels for the immunoblots presented in Figure 4a with HCC70 cells. Area cropped in indicated with red box, individual molecular weights of each individual antibody indicated on immunoblot.

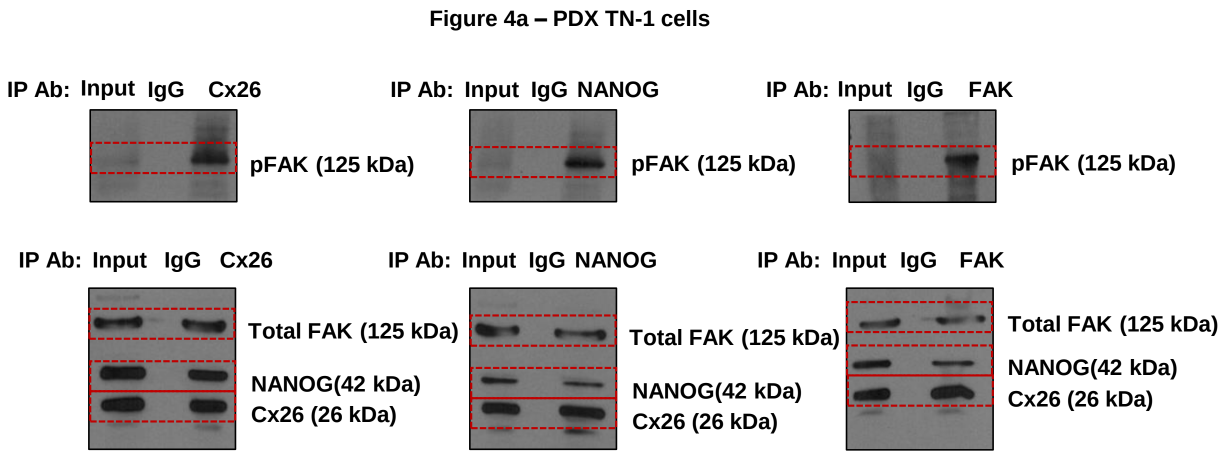


**Supplementary Figure 20. Original uncropped gels for PDX TN-1 cells in Figure 4a.** Original gels for the immunoblots presented in Figure 4a with PDX TN-1 cells. Area cropped in indicated with red box, individual molecular weights of each individual antibody indicated on immunoblot.

**Figure 4b – MCF7 cells**

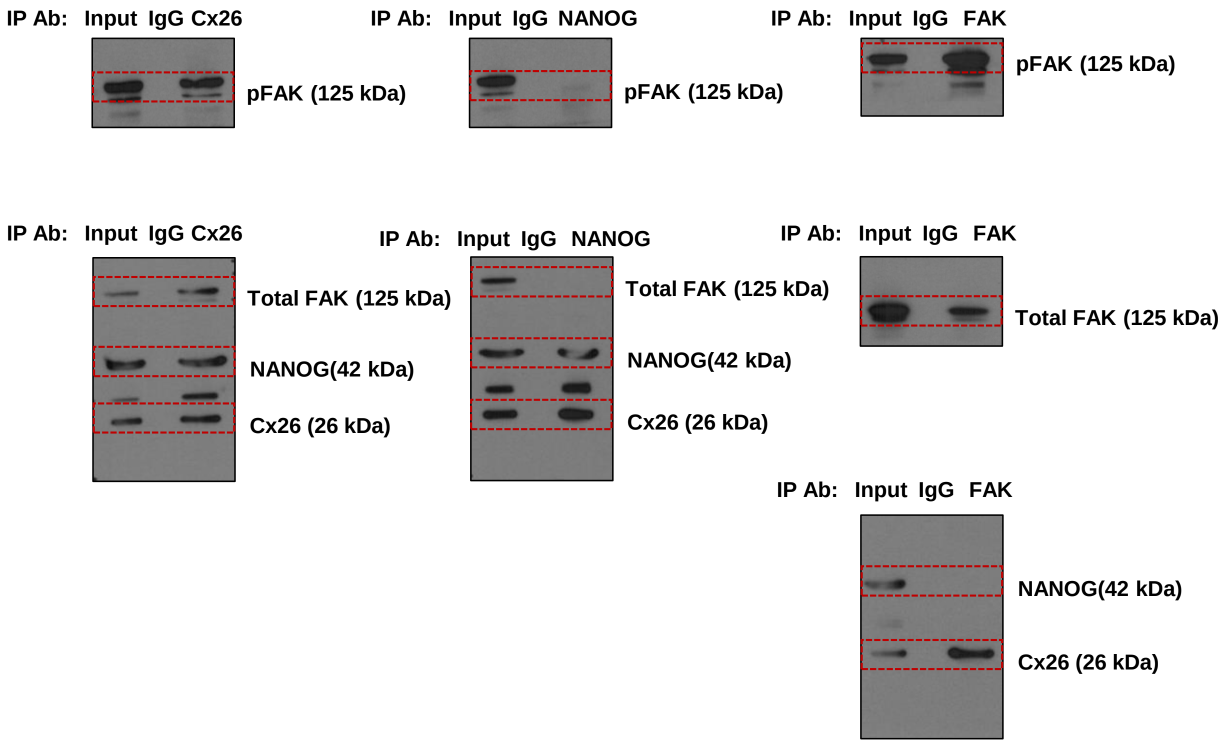


**Supplementary Figure 21. Original uncropped gels for MCF7 cells in Figure 4b.** Original gels for the immunoblots presented in Figure 4b with MCF7 cells. Area cropped in indicated with red box, individual molecular weights of each individual antibody indicated on immunoblot.

Figure 4b – MCF10A cells

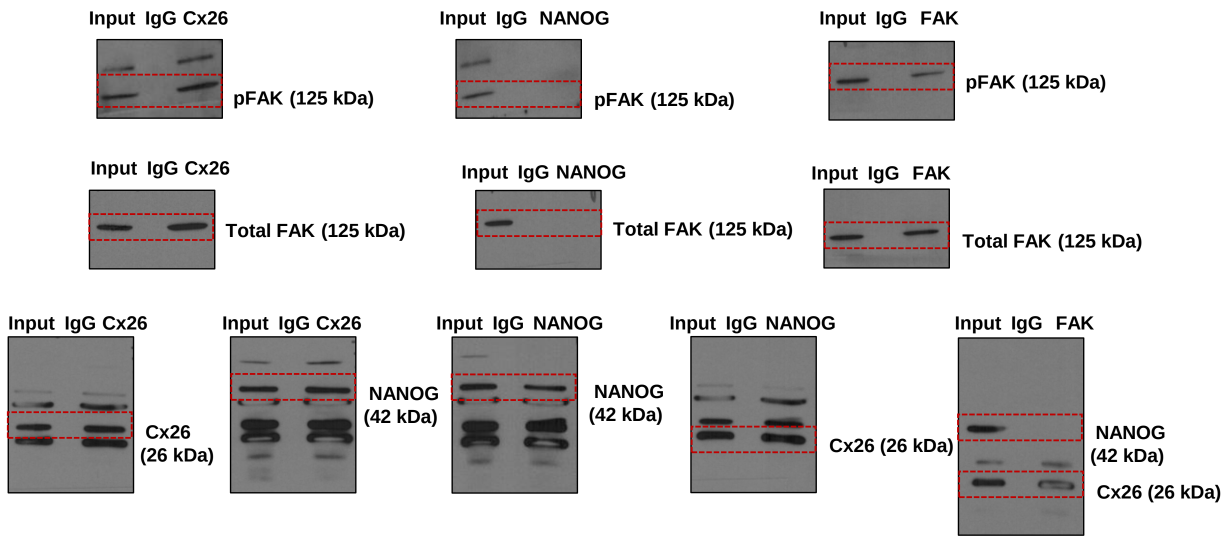


**Supplementary Figure 22. Original uncropped gels for MCF10A cells in Figure 4b.** Original gels for the immunoblots presented in Figure 4b with MCF10A cells. Area cropped in indicated with red box, individual molecular weights of each individual antibody indicated on immunoblot.

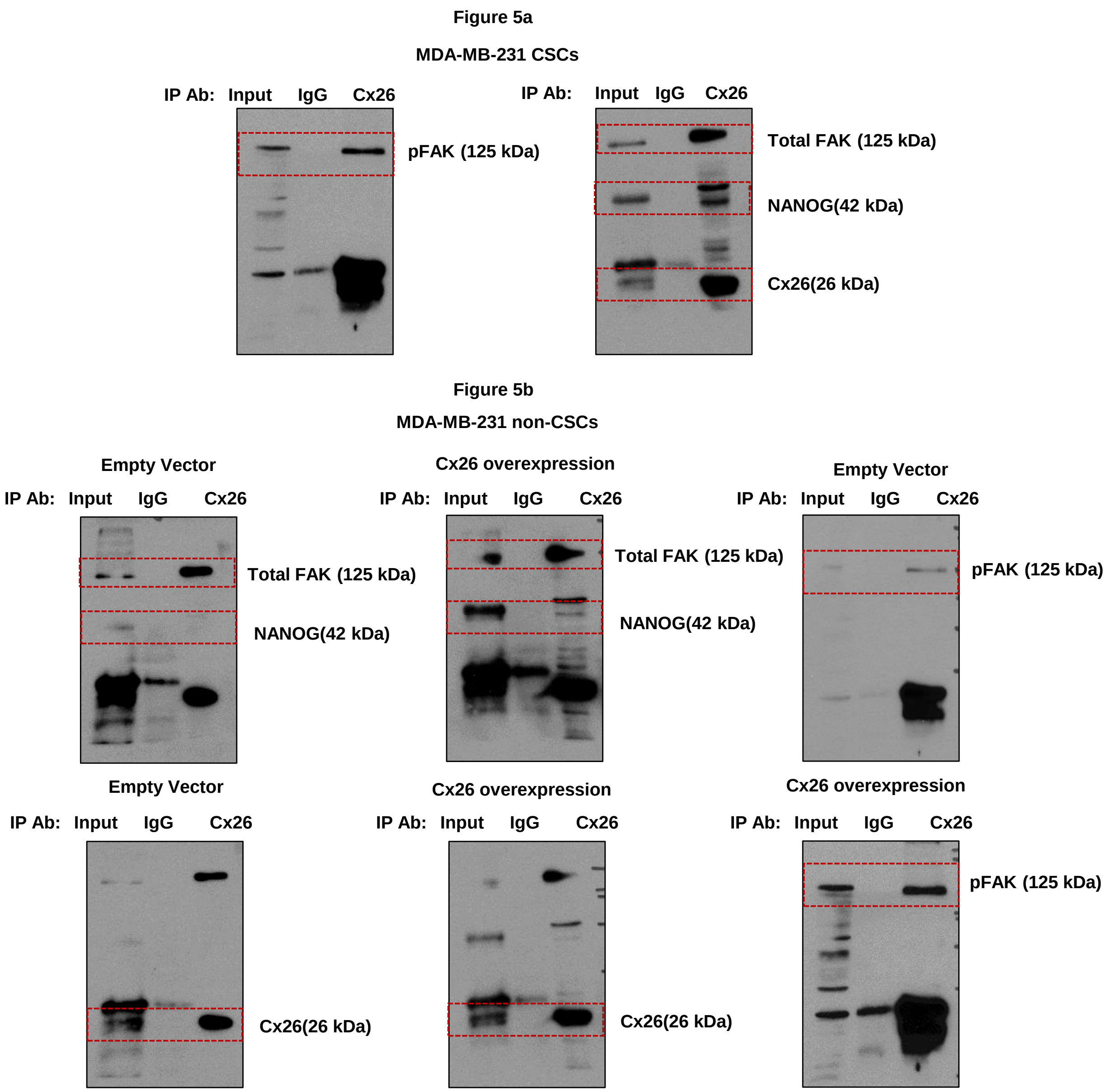


**Supplementary Figure 23. Original uncropped gels for Figure 5a and 5b.** Original gels for the immunoblots presented in Figure 5a and 5b. Area cropped in indicated with red box, individual molecular weights of each individual antibody indicated on immunoblot.

Figure 5c

MDA-MB-231 CSCs

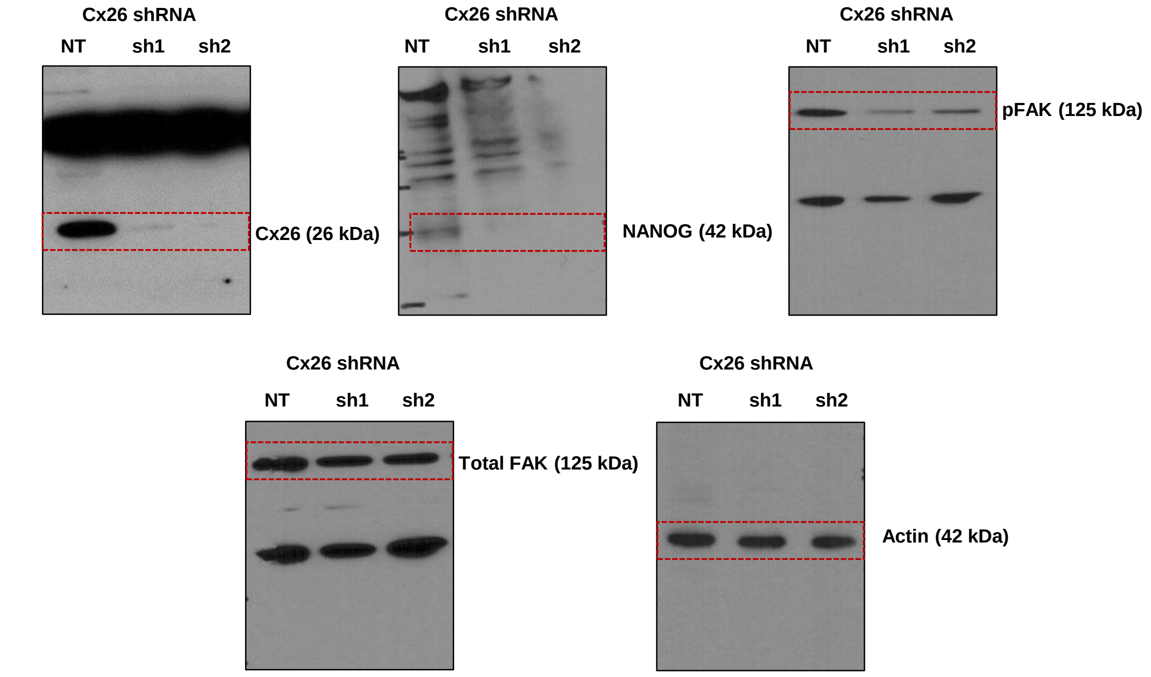


Figure 5e

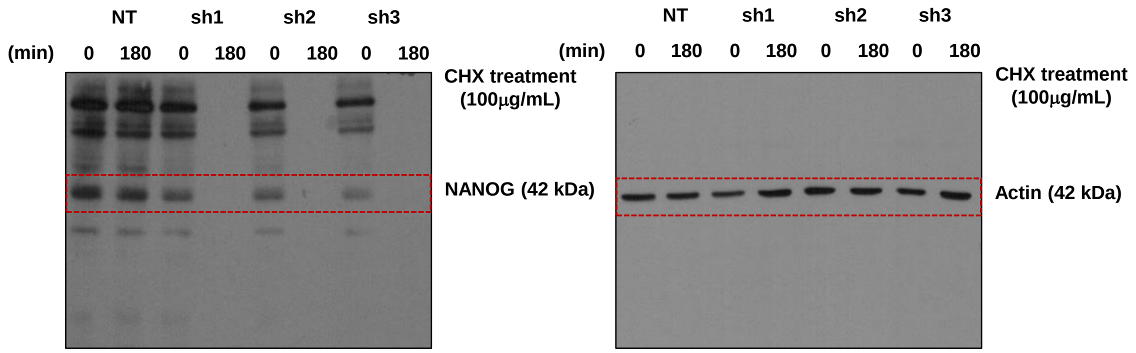


**Supplementary Figure 24. Original uncropped gels for Figure 5c and 5e.** Original gels for the immunoblots presented in Figure 5c and 5e. Area cropped in indicated with red box, individual molecular weights of each individual antibody indicated on immunoblot.

Figure 5f

MDA-MB-231 CSCs

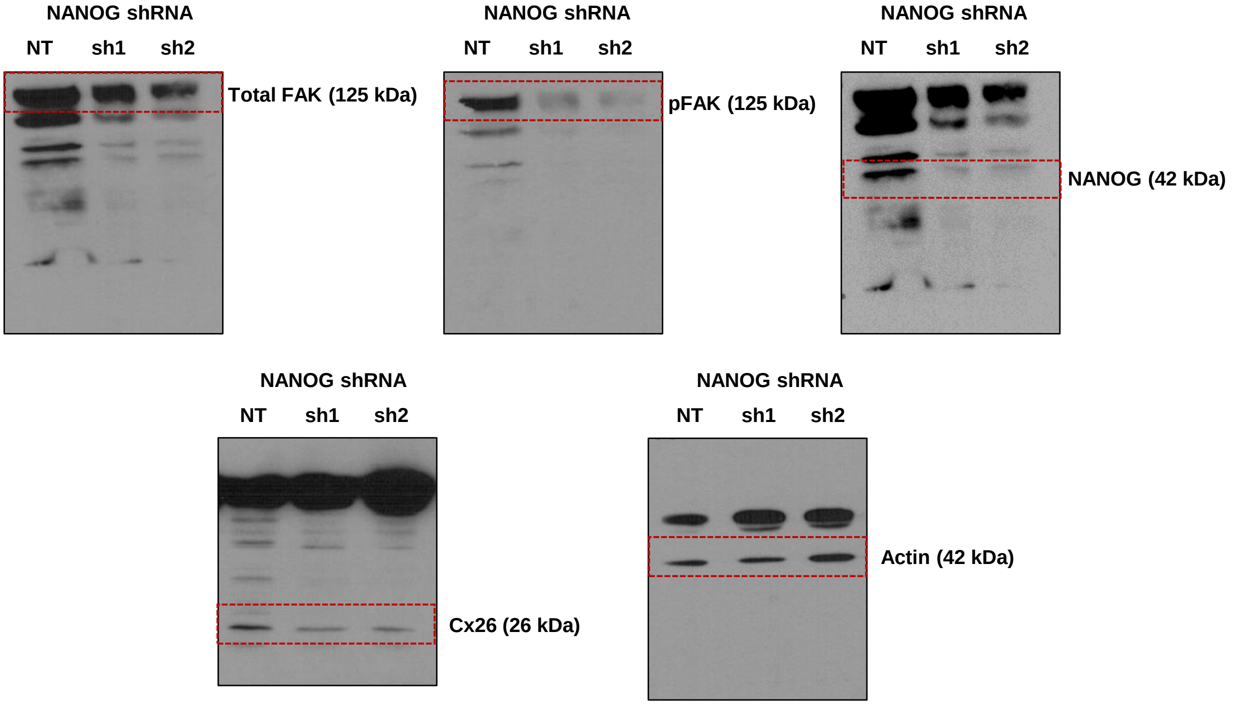


**Supplementary Figure 25. Original uncropped gels for Figure 5f.** Original gels for the immunoblots presented in Figure 5f. Area cropped in indicated with red box, individual molecular weights of each individual antibody indicated on immunoblot.

Figure 6a

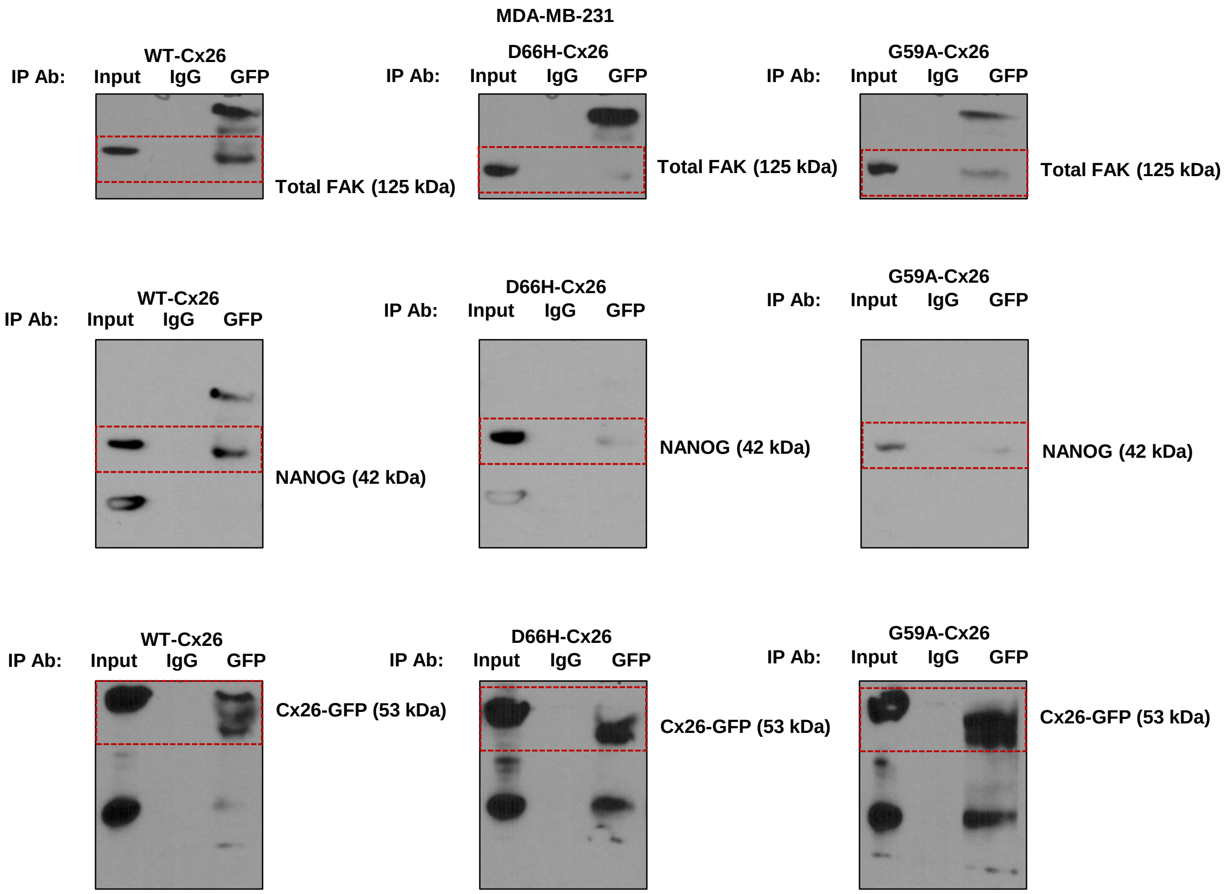


**Supplementary Figure 26. Original uncropped gels for Figure 6a.** Original gels for the immunoblots presented in Figure 6a. Area cropped in indicated with red box, individual molecular weights of each individual antibody indicated on immunoblot.

Figure 6b

PDX-TN-1

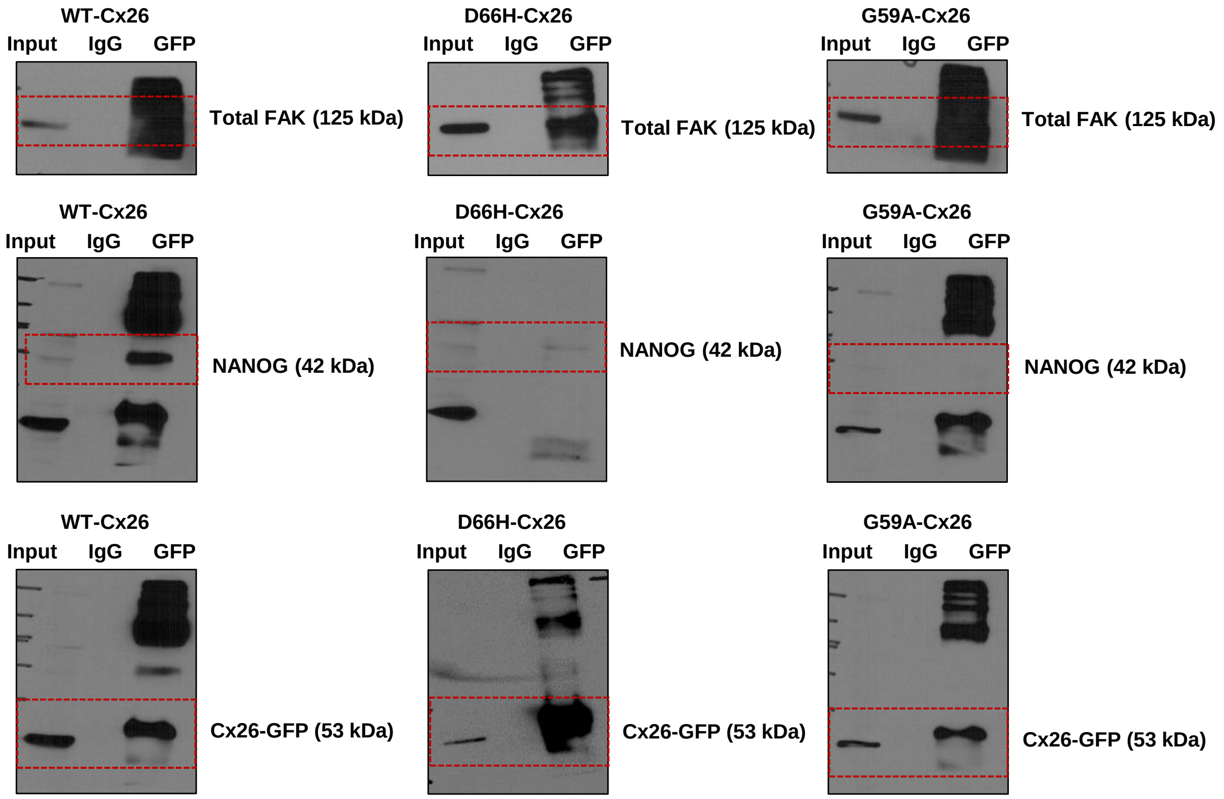


**Supplementary Figure 27. Original uncropped gels for Figure 6b.** Original gels for the immunoblots presented in Figure 6b. Area cropped in indicated with red box, individual molecular weights of each individual antibody indicated on immunoblot.

**Supplementary Figure 3c**

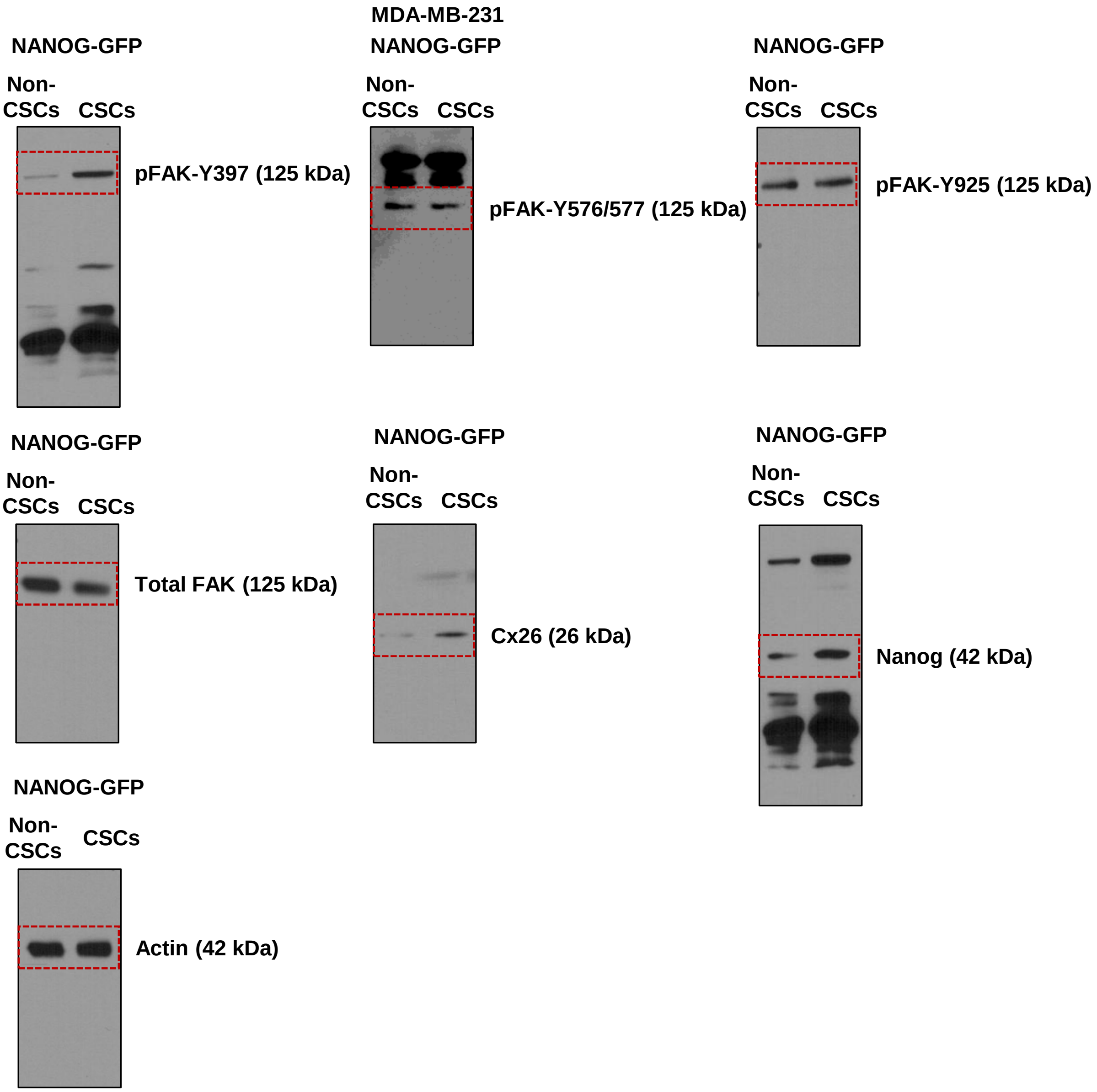


**Supplementary Figure 28. Original uncropped gels for Supplementary Figure 3c.** Original gels for the immunoblots presented in Supplementary Figure 3c. Area cropped in indicated with red box, individual molecular weights of each individual antibody indicated on immunoblot.

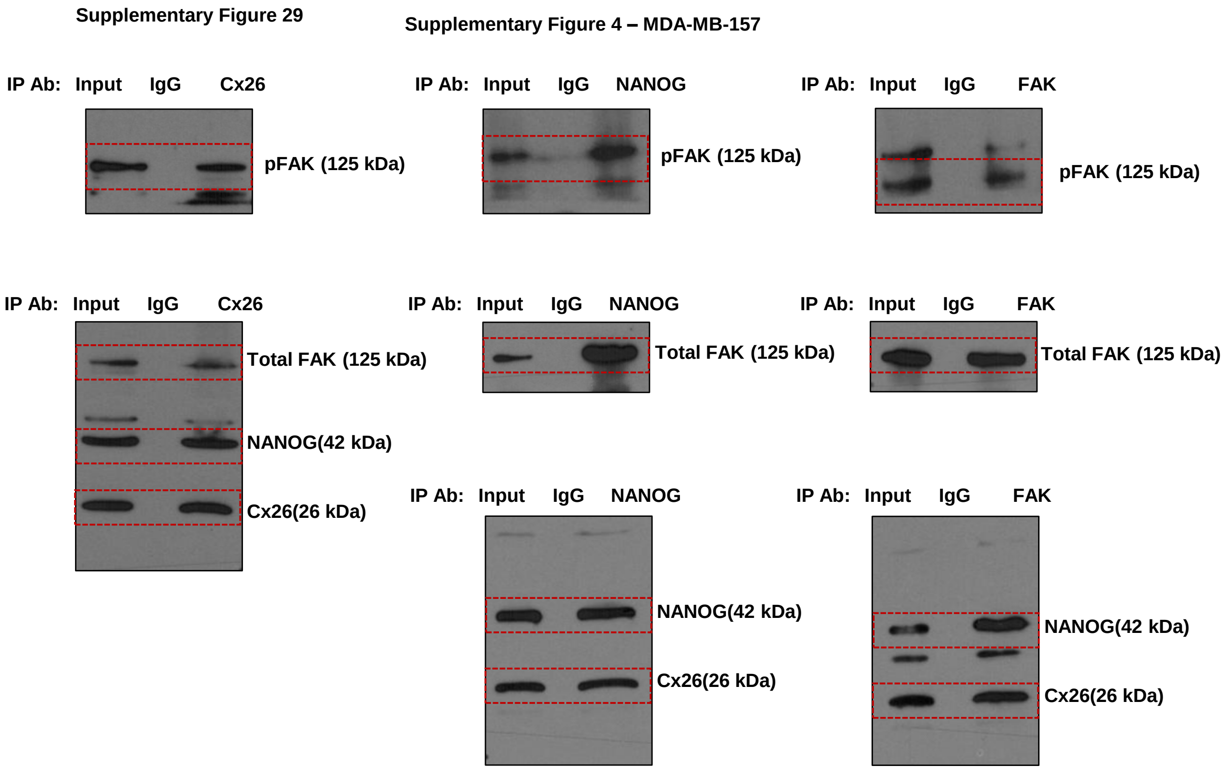


**Supplementary Figure 29. Original uncropped gels for MDA-MB-157 cells in Supplementary Figure 4.** Original gels for the immunoblots presented for MDA-MB-157 cells in Supplementary Figure 4. Area cropped in indicated with red box, individual molecular weights of each individual antibody indicated on immunoblot.

Supplementary Figure 4 – MDA-MB-468

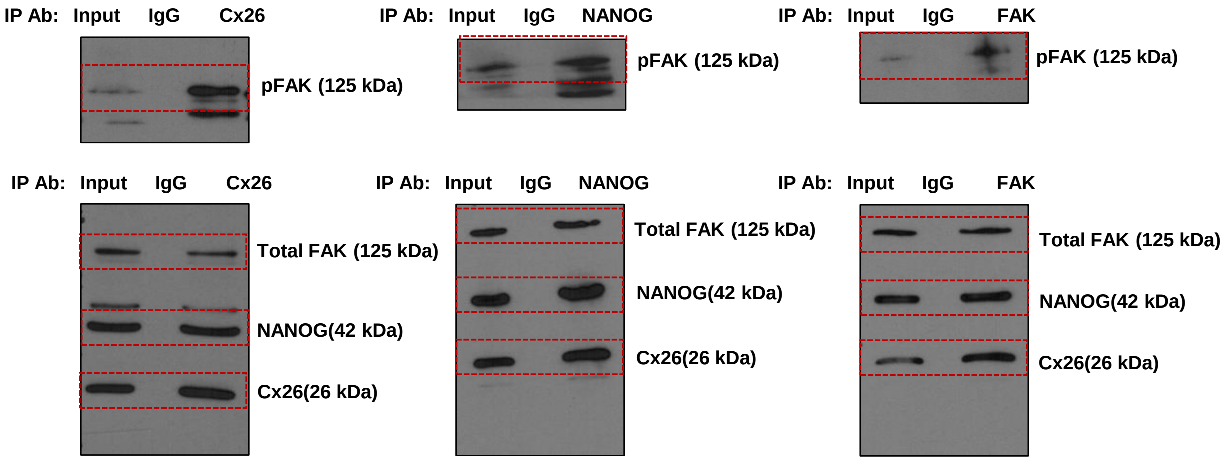


**Supplementary Figure 30. Original uncropped gels for MDA-MB-468 cells in Supplementary Figure 4.** Original gels for the immunoblots presented for MDA-MB-468 cells in Supplementary Figure 4. Area cropped in indicated with red box, individual molecular weights of each individual antibody indicated on immunoblot.

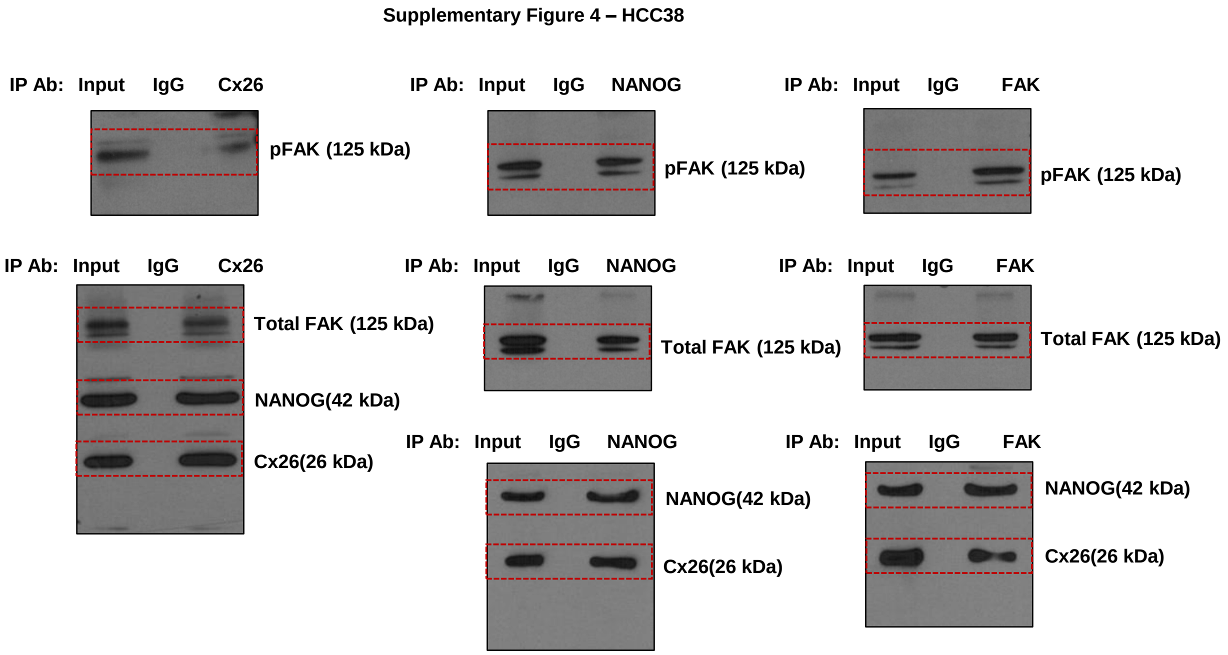


**Supplementary Figure 31. Original uncropped gels for HCC38 cells in Supplementary Figure 4.** Original gels for the immunoblots presented for HCC38 cells in Supplementary Figure 4. Area cropped in indicated with red box, individual molecular weights of each individual antibody indicated on immunoblot.

Supplementary Figure 5

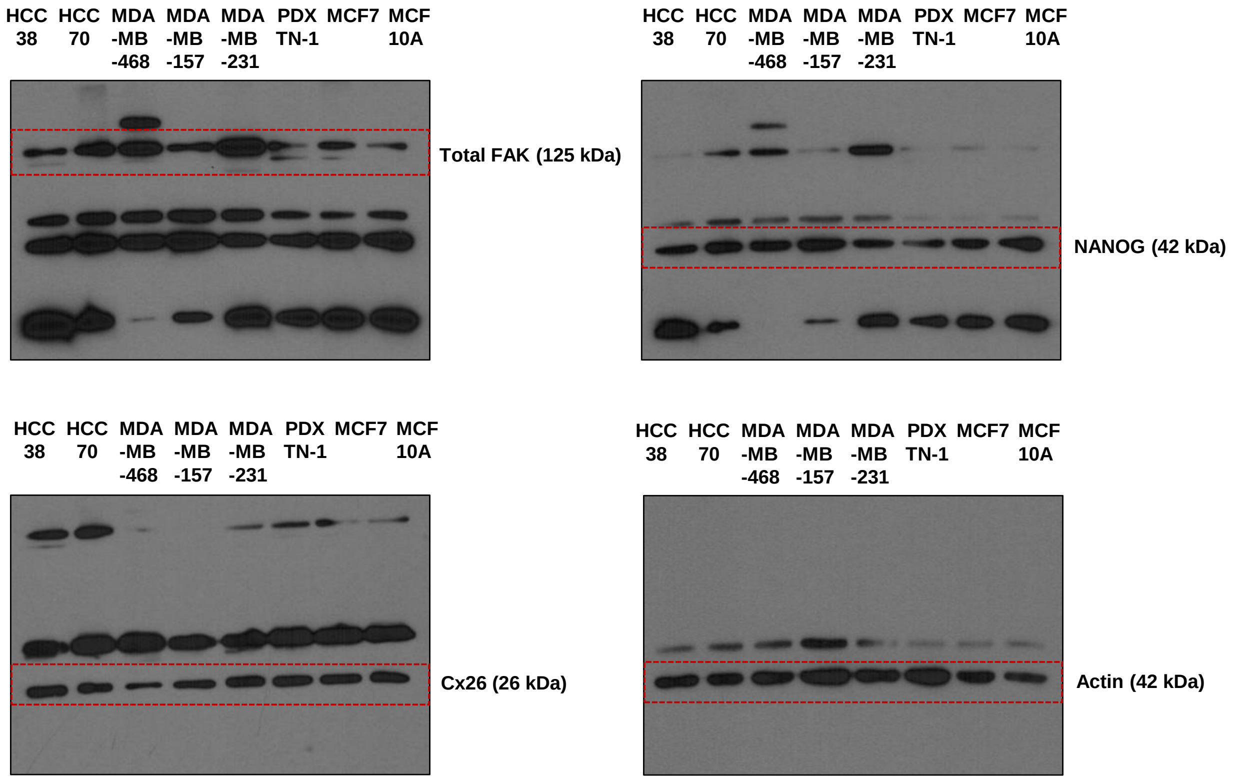


**Supplementary Figure 32. Original uncropped gels for Supplementary Figure 5.** Original gels for the immunoblots presented for Supplementary Figure 5. Area cropped in indicated with red box, individual molecular weights of each individual antibody indicated on immunoblot.

## Supplementary Figure 6a

### MDA-MB-231

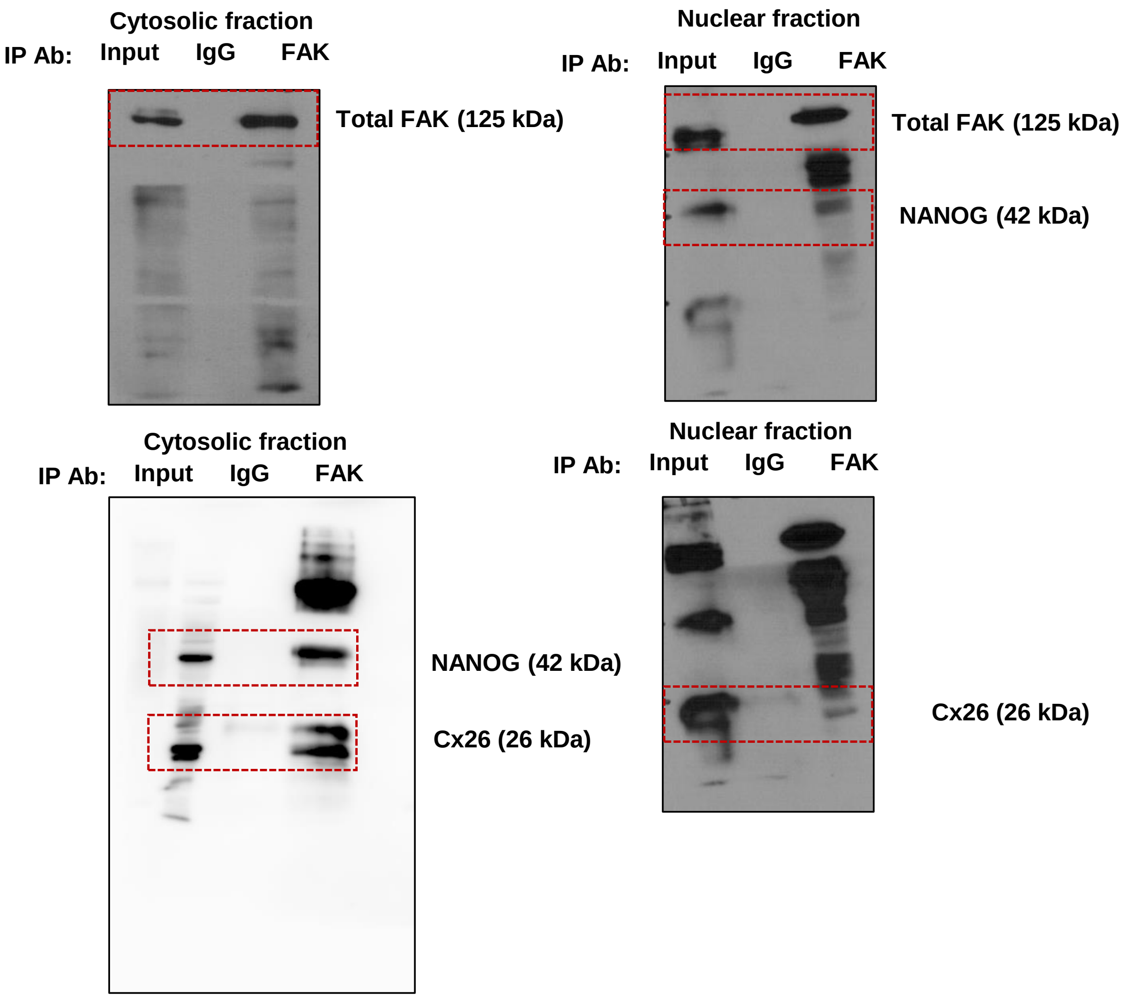


## Supplementary Figure 6b

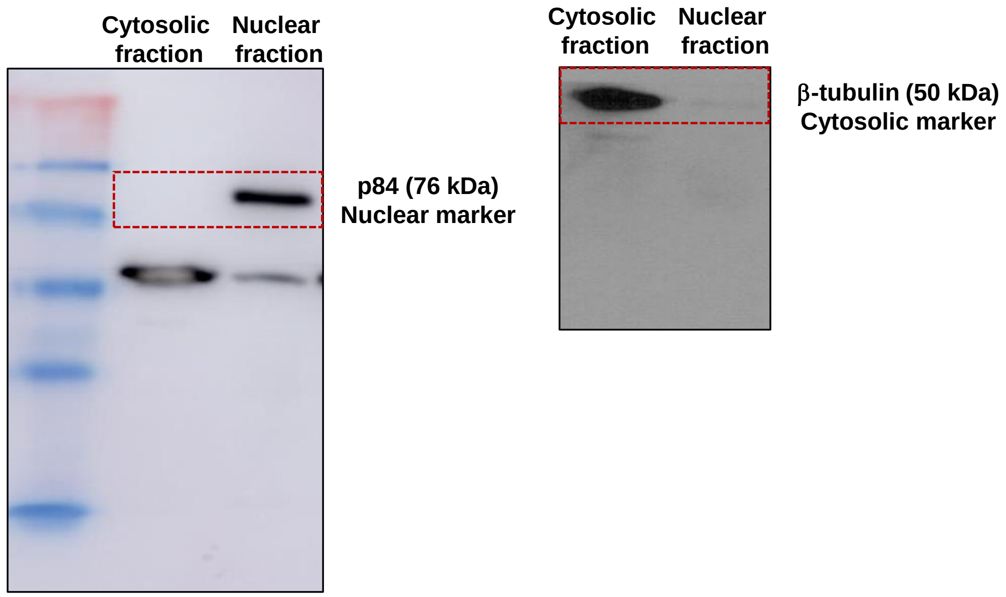

**Supplementary Figure 33. Original uncropped gels for Supplemental Figure 6a and 6b.** Original gels for the immunoblots presented for Supplemental Figure 6a and 6b. Area cropped in indicated with red box, individual molecular weights of each individual antibody indicated on immunoblot.

Supplementary Figure 7a

HCC70

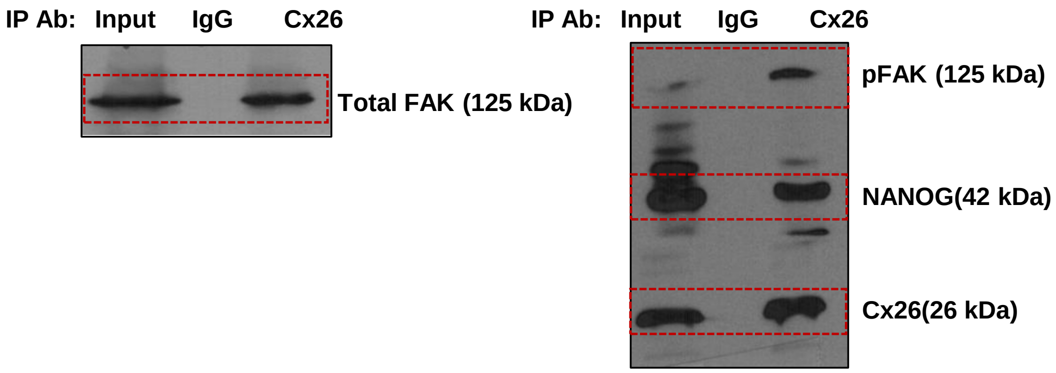


Supplementary Figure 7b

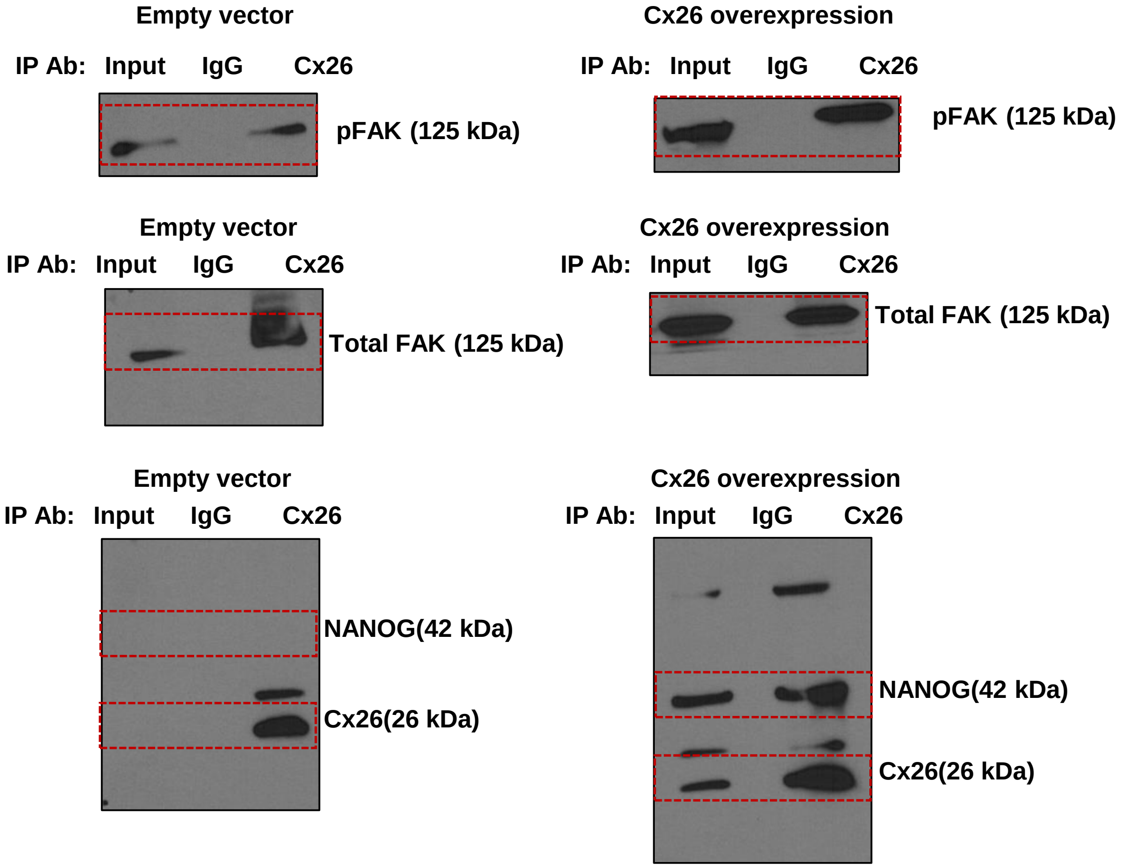


**Supplementary Figure 34. Original uncropped gels for Supplementary Figure 7a and 7b.** Original gels for the immunoblots presented for Supplementary Figure 7a and 7b. Area cropped in indicated with red box, individual molecular weights of each individual antibody indicated on immunoblot.

Supplementary Figure 7c

HCC70 CSCs

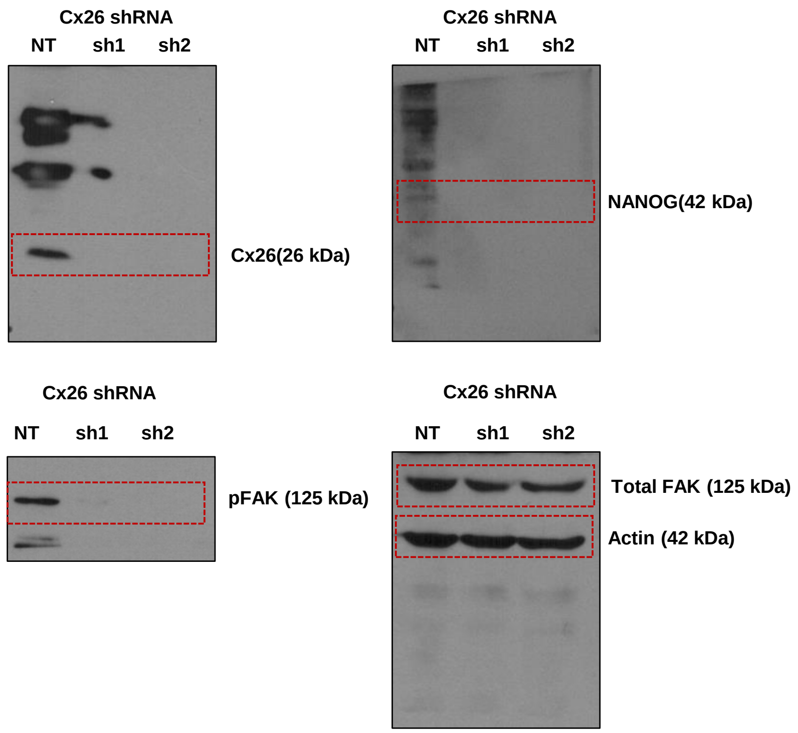


Supplementary Figure 7e

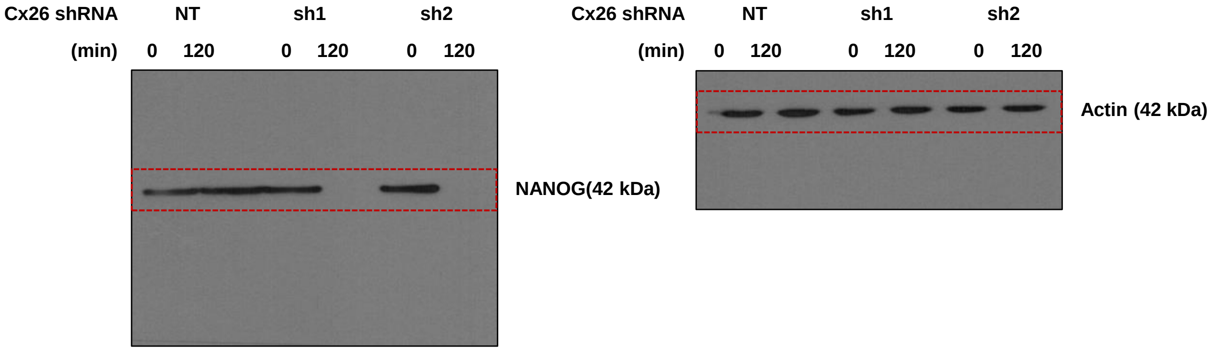


**Supplementary Figure 35. Original uncropped gels for Supplementary Figure 7c and 7e.** Original gels for the immunoblots presented for Supplementary Figure 7c and 7e. Area cropped in indicated with red box, individual molecular weights of each individual antibody indicated on immunoblot.

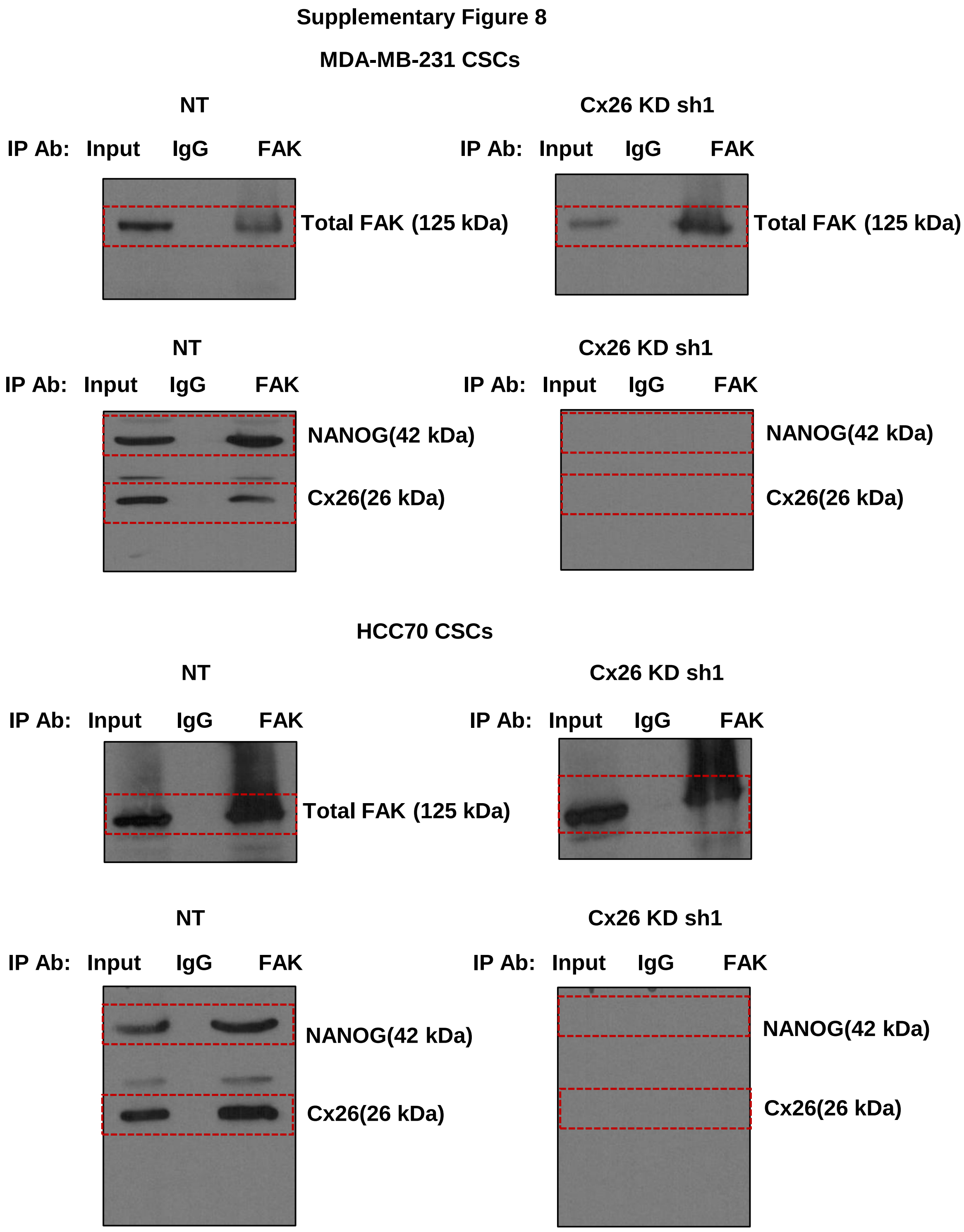


**Supplementary Figure 36. Original uncropped gels for Supplementary Figure 8.** Original gels for the immunoblots presented for Supplementary Figure 8. Area cropped in indicated with red box, individual molecular weights of each individual antibody indicated on immunoblot.

Supplementary Figure 9a

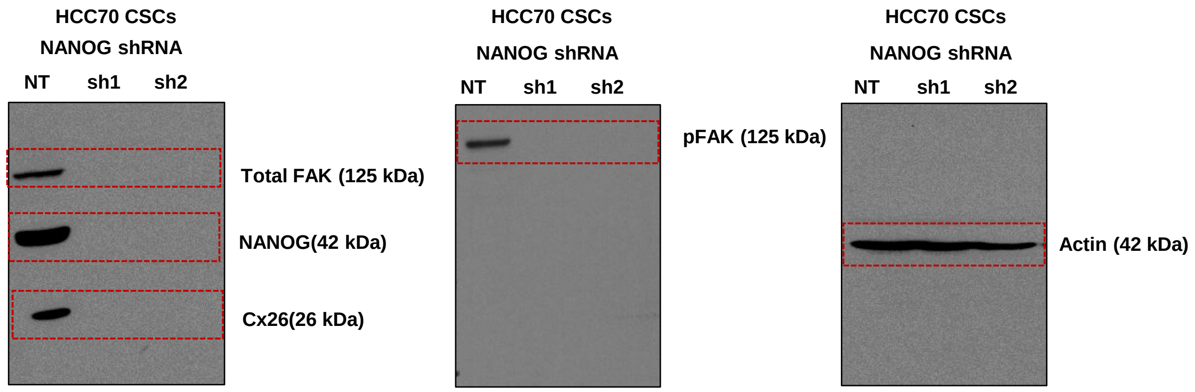


Supplementary Figure 10a

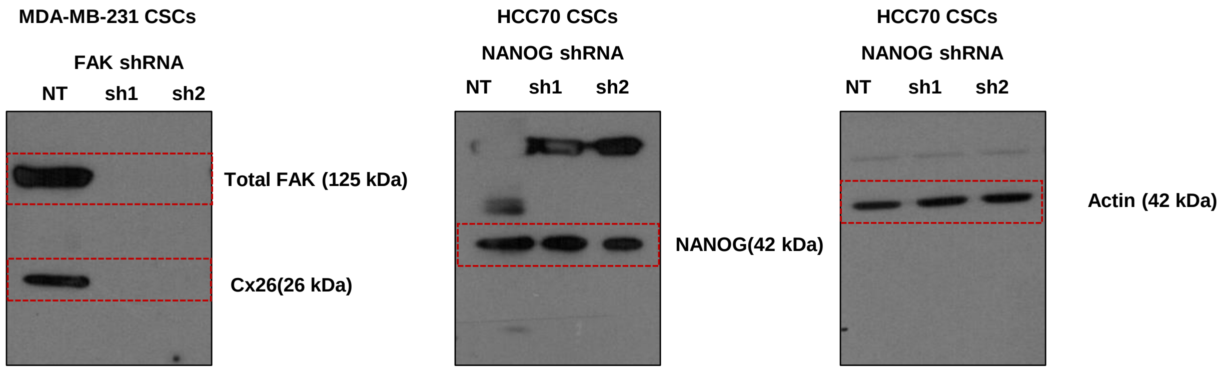


**Supplementary Figure 37. Original uncropped gels for Supplementary Figure 9a and 10a.** Original gels for the immunoblots presented for Supplementary Figure 9a and 10a. Area cropped in indicated with red box, individual molecular weights of each individual antibody indicated on immunoblot.

Supplementary Figure 10b

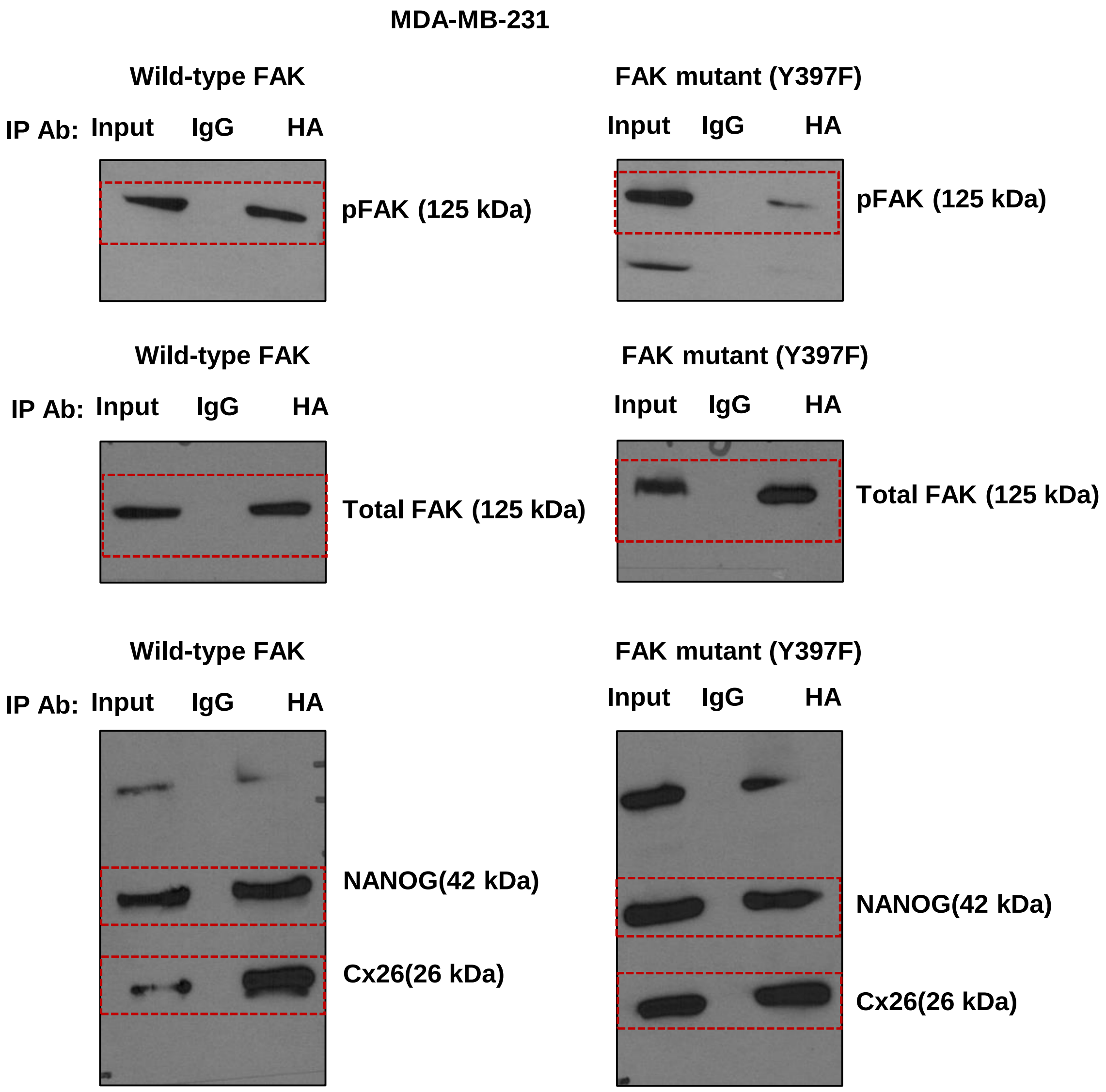


**Supplementary Figure 38. Original uncropped gels for Supplementary Figure 10b.** Original gels for the immunoblots presented for Supplementary Figure 10b. Area cropped in indicated with red box, individual molecular weights of each individual antibody indicated on immunoblot.

**Supplementary Table 1. Multivariate analysis of breast cancer and TNBC patient survival based on gene expression.** Data shown are median survival, n=3554 breast cancer patients, n=249 TNBC patients. Hazard ratios and *p*-values for individual and multiple gene combinations are indicated for breast cancer and TNBC patient survival.

| Gene | Tumor | Hazard ratio | p-value |
|---|---|---|---|
| *Cx43* | All breast cancer | 0.91 | 0.091 |
| *Cx46* | All breast cancer | 0.79 | <0.001 |
| *Cx26* | All breast cancer | 1.09 | 0.29 |
| *NANOG* | All breast cancer | 0.89 | <0.05 |
| *FAK* | All breast cancer | 1.04 | 0.53 |
| *Cx43/NANOG/FAK* | All breast cancer | 0.88 | 0.035 |
| *Cx46/NANOG/FAK* | All breast cancer | 1.02 | 0.74 |
| *Cx26/NANOG/FAK* | All breast cancer | 0.97 | 0.7 |
| *Cx43* | TNBC | 1.31 | 0.28 |
| *Cx46* | TNBC | 1.29 | 0.3 |
| *Cx26* | TNBC | 1.74 | 0.09 |
| *NANOG* | TNBC | 0.86 | 0.49 |
| *FAK* | TNBC | 1.14 | 0.55 |
| *Cx43/NANOG/FAK* | TNBC | 1.45 | 0.13 |
| *Cx46/NANOG/FAK* | TNBC | 1.40 | 0.17 |
| ***Cx26/NANOG/FAK*** | **TNBC** | **2.49** | **<0.01** |
| *NANOG/FAK* | TNBC | 1.4 | 0.18 |

**Supplementary Table 2. Primer sequences used for quantitative real-time PCR analyses**

| Gene | Direction | Sequence |
|---|---|---|
| *β-Actin* | Forward | 5'-AGAAAATCTGGCACCACACC-3' |
| | Reverse | 5'-AGAGGCGTACAGGGATAGCA-3' |
| *NANOG* | Forward | 5'-CCCAAAGGCAAACAACCCACTTCT-3' |
| | Reverse | 5'-AGCTGGGTGGAAGAGAACACAGTT-3' |
| *Cx26* | Forward | 5'-TTAAAAGGCGCCACGGCGGGA-3' |
| | Reverse | 5'-ATCCATCTTCTACTCTGGGC-3' |
