# Supplementary material for: Cx26 drives self-renewal in triple-negative breast cancer via interaction with NANOG and focal adhesion kinase
Source: Nat Commun. 2018 Feb 8;9:578. doi: 10.1038/s41467-018-02938-1 (PMC5805730; doi:10.1038/s41467-018-02938-1)
Supplement: Supplementary file 2 — Description of Additional Supplementary Files [file 41467_2018_2938_MOESM2_ESM.pdf]

Description of Additional Supplementary Files

File Name: Supplementary Movie 1
Description: Time-lapse video clip demonstrating limited dye transfer in MDA-MB-231 CSCs injected with IgG control and biocytin-rhodamine dye.
